# The art of falling: identifying the falls scenarios associated with bouldering injuries

**DOI:** 10.3389/fspor.2025.1609133

**Published:** 2025-07-17

**Authors:** Erwan Beurienne, Nicolas Bailly, Maxime Luiggi, Cécile Martha, Claire Bruna-Rosso, Maxime Wylomanski, Michel Behr, Marine Dorsemaine

**Affiliations:** ^1^ Laboratoire de Biomécanique Appliquée, Aix Marseille Université/Université Gustave Eiffel, Marseille, France; ^2^ Tyyny, Context’, Le Chambon-Feugerolles, France; ^3^ ADEF, Aix Marseille Université, Marseille, France; ^4^ INSERM, IRD, SESSTIM, Sciences Economiques & Sociales de la Santé & Traitement de l'Information Médical, ISSPAM, Aix Marseille Université, Marseille, France; ^5^ ISM, CNRS, Aix Marseille Université, Marseille, France

**Keywords:** climbing, bouldering, injury, fall, kinematics, pads

## Abstract

**Introduction:**

Bouldering has seen a significant rise in popularity, accompanied by an increase in related injuries, primarily caused by falls. To enhance prevention strategies and improve protective mats, it is crucial to understand the mechanisms behind these injuries. However, there is limited knowledge about the specific fall scenarios leading to bouldering injuries. The aim of this study was to provide a detailed description of accident scenarios and fall kinematics leading to bouldering injuries.

**Methods:**

Adults (aged 18 and above) who experienced an acute fall-related injury while bouldering were invited to complete a self-reported online survey. They were recruited between February 2024 and March 2025 through emails and QR-code posters via university mailing lists and partnerships with French bouldering gym franchises. A total of 245 participants provided personal information, descriptions of their injuries, details about the climbing routes, and fall scenarios, including the kinematics of the fall.

**Results:**

A majority of the injuries affected the lower limb (67% of the case) with ankle sprain being the primary injury. Most of the falls (85%) were involuntary with 62% of them starting in a vertical position, frequently followed by a rotation during the fall (62%), and resulted in a feet first landing (74%). Most injuries happened after falls from vertical walls (45%) and steep walls (29%), primarily from the middle (32%) and the top (47%) sections of the wall.

**Conclusion:**

These results provide a first detailed description of the fall scenarios associated with injuries in bouldering and show that different injury mechanisms (such as vertical versus leaning positions during a fall) result in distinct patterns of injury. Such findings could be used to develop new pads with higher density or multi-layering, or to devise safer falling techniques that can be taught by trainers.

## 1 Introduction

Climbing, and especially bouldering, has seen a significant rise in popularity over the last decade. In France, the number of climbers grew from 1 to 3 million between 2016 and 2020, while climbing gym attendance increased by 29% between 2019 and 2023 (1, 2). Similar trends are observed in Germany, United States, and Canada (3–7).

Concurrently, there has been an increase in climbing-related injuries. In the US, national rock climbing injury estimates nearly doubled between 2008 and 2016 (8). A similar trend was observed in bouldering, where the number of injuries increased from 3 to 71 cases between 2010 and 2018 among patients presenting at a German level I trauma unit (9). Besides, bouldering was identified as the climbing discipline most prone to accidents, with a higher number of injuries reported compared to other climbing disciplines. This finding is supported by a range of evidence, including questionnaire data (10, 11), clinical records (12, 13), and German climbing gym statistics, which show that bouldering-related injuries account for 71% of all injuries in their gyms (14).

Bouldering, a discipline of climbing, typically involves scaling short walls around 4.5 meters high without the use of ropes. Injuries in this sport generally fall into two categories: overuse and acute injuries. Overuse injuries correspond to chronic injuries without a singular causing event and primarily affect the upper limb (15–19). In contrast, acute injuries are often linked to fall-related injuries, primarily affect the lower limb (8, 9, 20–22), and tend to be more severe than overuse injuries (8, 9, 15, 19). These discrepancies among studies can be attributed to differences in study design (9). Since falls are inherent to bouldering, as the sport does not use ropes or harnesses, it is essential to protect climbers from them. In that aim, 30 or 40 cm thick foam pads are typically placed on the floor to provide a safer landing surface. However, to further reduce the risk of fall-related injuries, there are two potential areas for improvement: enhancing the performance of existing safety pads to reduce the injury risk and implementing preventive measures such as spotting or safe falling techniques. However, to inform the design of effective pads and preventive measures, it is essential to better understand how falls lead to injury (fall scenarios, fall kinematics, impact velocity, risk factor etc.) (23). Unfortunately, current research on bouldering accidentology primarily focuses on injury descriptions (localization, type, severity) and potential risk factors (gender, skill level, injury history, body weight, etc.), without examining the accident scenario and injury mechanism in detail. Although fall mechanisms have been investigated in the context of rock climbing (24), these mechanisms are distinct from those observed in bouldering, primarily due to the use of ropes. Then, the aim of this study is to provide a detailed description of the accident scenarios and the fall kinematics leading to bouldering injuries.

## 2 Materials and methods

### 2.1 Study design

A retrospective online survey was distributed to French-speaking climbers who sustained a fall-related injury during bouldering. An injury was defined as any musculoskeletal complaint or pain that altered the usual mode, duration, intensity, or frequency of training or competitions in accordance with the IOC consensus statement (25). Participants were recruited between February 2024 and March 2025 through emails and posters with QR codes using two channels. First, emails were sent via existing mailing lists to personnel and students at Aix-Marseille University and Gustave Eiffel University. Second, the survey was shared via email and QR-code posters in partnership with two French bouldering gym franchises. No additional incentives were provided to encourage participation. The study included all individuals over 18 years of age who self-declared a fall-related injury while bouldering. Participants with more than 10% of missing responses, based on the question they were asked, were excluded from the study. Furthermore, during the process of data analysis, the consistency of participants' responses was examined, particularly those with “other” categories (questions where participants could select “other” and fill in a free-text field). If a participant's responses did not meet the inclusion criteria (e.g., the respondent is under 18 or has fallen while bouldering outdoors), they were removed from the study. Prior to data collection, the study protocol was approved by the Ethics Committee of Gustave Eiffel University.

### 2.2 Survey

**Figure 1:**
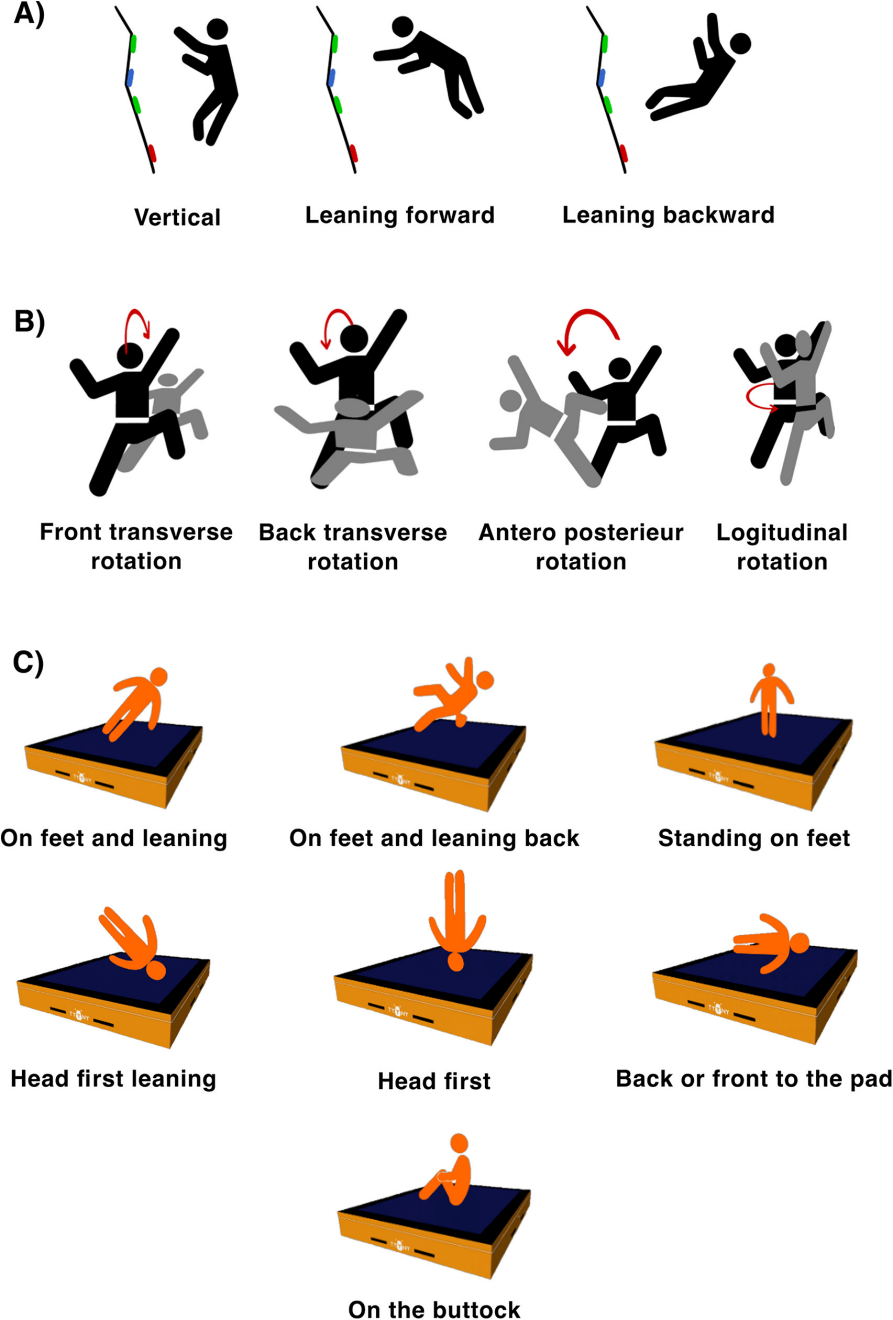
Schematic representation of fall kinematics for **(A)** the position start fall, **(B)** the rotation during the fall and **(C)** the position at landing.

The survey, constructed on the basis of previous studies (9, 15, 16), was divided into three parts. The first part gathered general information about the participant, including sex, age, skill level [represented by the usual difficulty of routes climbed after work using Bleau rating (16) and number of bouldering sessions per week] and history of bouldering fall-related injuries. The second part focused on the injury resulting from the accident, including information about the injury's location using the OSICS anatomical site code (26), the type of injury, and the severity of the injury using the UIAA classification (27) and the time lost due to the injury. The last part focused on the fall scenario: route information (wall type, difficulty, fall height), reason for the fall (voluntary or involuntary, during static or dynamic movement, cause of the fall), fall kinematics [position start fall (Figure 1A), rotation during the fall (Figure 1B), and position at landing (Figure 1C)], and the impact (landing surface). Prior to the general distribution of the survey, cognitive interviews were conducted with students from the sport faculty reporting a climbing fall related injury to ensure the clarity and relevance of the questions, and to estimate the average time required to complete the questionnaire. The complete survey is available in the Supplementary Materials.

### 2.3 Data analysis

Numerical variables such as age and time loss were recategorized. Age was divided into four groups based on quantile distribution: 18–23 years, 24–27 years, 28–31 years and 32–58 years. Time loss was categorized into three intervals: 1–7 days, 8–28 days, and more than 28 days according to IOC consensus statement (25). The number of bouldering training sessions per week were grouped as 1 training, 2 training and more than 2 training per week. The injury severity variable was adjusted by combining the UIAA 4 and UIAA 3 modalities into a single category labeled UIAA 3+, due to only one respondent reporting an injury UIAA 4. Finally, injury localization responses were group in head/neck, upper limb, trunk, lower limb categories to increase group size during analysis.

Descriptive statistics, along with 95% confidence intervals, were calculated using the Wilson score interval method. Fall scenarios were analyzed according to injury location and type.

A multivariate logistic regression analysis was conducted to identify the factors influencing the severity of injuries. The outcome variable, injury severity, was categorized into two levels: “minor injury” (corresponding to UIAA 1 and 2) and “severe injury” (corresponding to UIAA 3 and 4). The following predictor variables were included in the model: age; sex; skill level; fall height; wall type; and fall kinematics (encompassing position at the start of the fall, rotation during the fall, and landing position). Among these predictor variables, only the modalities with at least 10 occurrences were retained for the analysis (28). To ensure a consistent reference point, the modality with the highest frequency of occurrence was designated as the reference category for each variable. All data processing and analysis were conducted using R (version 4.4.2, R Core Team).

## 3 Results

A total of 402 climbers responded to the survey. Among these participants, 135 were excluded due to a high rate of missing values among their responses. An additional 15 responses were excluded because they were outside the study's scope, including accidents during lead climbing or injuries not caused by falls. Furthermore, 7 participants were under 18 years old. Finally, 245 responses were analyzed, corresponding to 301 injuries, with 56 participants reporting multiple injuries.

### 3.1 Data description

#### 3.1.1 Population

The study participants were predominantly female (62%), with an average age of 29.2 ± 7.7 years (Table 1). Most of the participants reported a skill level between 7a and 7b + (44%) or between 6a and 6c + (42%) and boulderer 1 (47%) to 2 times a week (33%). Notably, 27% of respondents reported experiencing anterior bouldering fall-related injuries. Among those injured, 49% sustained 1 injury, 24% experience 2 injuries, and 24% had more than 2 injuries.

**Table 1:** Climbers profile and injury description.

Variables	Modalities	*n* (%)	CI 95%
Climber information			
Sex	Female	151 (62%)	55.4–67.5
	Male	89 (36%)	30.6–42.5
	Unknown	5 (2%)	0.9–4.7
Age	18–23	58 (24%)	18.8–29.4
	24–27	62 (25%)	20.3–31.1
	28–31	54 (22%)	17.3–27.6
	32–58	70 (29%)	23.3–34.5
	Mean ± std	29.2 ± 7.7	–
Skill level	Easy (1–5+)	18 (7%)	4.7–11.3
	Medium (6a–6c+)	102 (42%)	35.6–47.9
	Hard (7a–7b+)	107 (44%)	37.6–49.9
	Very hard (7c et +)	15 (6%)	3.7–9.9
	Unknown	3 (1%)	0.4–3.5
History of injury	Yes	67 (27%)	22.1–33.2
	No	172 (70%)	64.2–75.6
	Unknown	6 (3%)	1.1–5.2
Number of bouldering training per week	1	100 (41%)	34.8–47.1
	2	80 (33%)	27.1–38.8
	More than 2	64 (26%)	21–32
	Unknown	1 (>1%)	0.1–2.3
	Mean ± sd	1.8 ± 1.1	–
Injury			
Injury location	Head/Neck	10 (3%)	1.8–6.0
	Upper limb	76 (25%)	20.7–30.4
	Trunk	12 (4%)	2.3–6.8
	Lower limb	201 (67%)	61.3–71.9
	Unknown	2 (1%)	0.2–2.4
Injury type	Sprain	107 (36%)	30.4–41.1
	Fracture	68 (23%)	18.2–27.6
	Dislocation	34 (11%)	8.2–15.4
	Ligament/tendon rupture	33 (11%)	7.9–15
	Tendonitis	7 (2%)	1.1–4.7
	TBI	4 (1%)	0.5–3.4
	Bruises	9 (3%)	1.6–5.6
	Cutaneous injury	4 (1%)	0.5–3.4
	Other	21 (7%)	4.6–10.4
	Unknown	14 (5%)	2.8–7.7
Injury severity	UIAA 1	42 (14%)	10.5–18.3
	UIAA 2	192 (64%)	58.9–69.6
	UIAA3+	59 (20%)	15.5–24.4
	Unknown	6 (2%)	0.9–4.3
Time loss from climbing	1–7 days	6 (3.4%)	1.6–7.4
	8–28 days	15 (9%)	5.3–13.8
	>28 days	152 (88%)	82.2–91.9
	Still injured	72 (− %)	–
	Mean ± sd	132.4 ± 143	–

#### 3.1.2 Injuries

Injuries mostly occurred in the lower limbs (67%) and on the upper limbs (25%), with the ankle (40%), the elbow (16%) and the knee (15%) being the most injured body parts (Table 1). Sprains were the most frequent type of injury (36%), followed by fractures (23%), dislocations (11%), and tendon or ligament ruptures (11%). Specifically, ankle sprains were the most prevalent specific injury (28%), followed by ankle fractures (8%) and elbow dislocations (7%). Injuries were mostly UIAA 2 (64%), UIAA 3+ (20%) or UIAA 1 (14%) and the severity of injuries was consistent across all body locations, with an average UIAA of 1.6 (calculated using the numerical part of the UIAA scale). The median [Q1; Q3] time without climbing after an injury was 90 [50; 180] days, with the shortest duration being 5 days and the longest 900 days. This median time lost was the same for lower and upper limbs injuries: 90 [60; 180] days.

Table 2 presents the distribution of injury locations according to injury type (restricted to the four main injuries) and time lost from climbing (excluding respondents still injured). Sprains and tendon or ligament ruptures primarily affected the lower limbs, with 93% and 82% of these injuries occurring there, respectively. Fractures also predominantly affected the lower limbs, though to a lesser extent (71%). Conversely, dislocations primarily occurred in the upper limbs (85%) (Table 2).

**Table 2:** Descriptive presentation of the 4 main types of injury and the severity of injury (as time not climbing due to injury) as function of the location of injury, grouped into 4 categories.

Location	Head/Neck		Upper limb		Trunk		Lower limb	
	*n* (%)	95% CI	*n* (%)	95% CI	*n* (%)	95% CI	*n* (%)	95% CI
Type								
Sprain	2 (2%)	0.5–6.6	6 (6%)	2.6–11.7	0 (0%)	–	99 (93%)	85.9–96.2
Fracture	0 (0%)	–	18 (26%)	17.4–38	2 (3%)	0.8–10.1	48 (71%)	58.9–80.1
Dislocation	0 (0%)	–	29 (85%)	69.9–93.6	0 (0%)	–	5 (15%)	6.4–30.1
Tendon or ligament rupture	0 (0%)	–	6 (18%)	8.6–34.4	0 (0%)	–	27 (82%)	65.6–91.4
Total	2 (1%)	0.2–3.0	59 (24%)	19.4–30.2	2 (1%)	0.2–3.0	179 (74%)	68.1–79.1
Time loss from climbing								
1–7 days	2 (33%)	9.7–70	0 (0%)	–	0 (0%)	–	4 (67%)	30–90.3
8–28 days	2 (13%)	3.7–37.9	2 (13%)	3.7–37.9	1 (7%)	1.2–29.8	10 (67%)	41.7–84.8
>28 days	3 (2%)	0.7–5.6	39 (26%)	19.4–33.1	5 (3%)	1.4–7.5	105 (69%)	61.3–75.9
Total	7 (4%)	2.0–8.1	41 (24%)	18.0–30.6	6 (4%)	1.6–7.4	119 (69%)	61.5–75.2

#### 3.1.3 Fall scenarios

Most injuries resulted from involuntary falls (85%), occurring after either static (41%) or dynamic (41%) movements. The three most common causes of falls were foot slips, missed holds, and dynos, accounting for 72% of all accidents. In 94% of the accidents, the climber landed on a safety pad. Vertical and steep walls were the most accident-prone walls (74%) and falls at the top of the wall were the most frequent (47%). At the time of the accident, 58% of participants were climbing a route at their skill level. Regarding the movements during the fall, 62% of accidents began from a vertical position, 62% involved a rotation, and 73% ended with the climber landing on their feet (Table 3).

**Table 3:** Fall scenarios description.

Variables	Modalities	*n* (%)	CI 95%
Route information			
Wall type	Vertical	110 (45%)	38.8–51.2
	Steep	72 (29%)	24–35.4
	Slab	29 (12%)	8.4–16.5
	Roof/overhang	17 (7%)	4.4–10.8
	Corner	12 (5%)	2.8–8.4
	Unknown	5 (2%)	0.8–4.7
Route difficulty	Way below my level	5 (2%)	0.9–4.7
	Below my level	22 (9%)	6–13.2
	At my level	142 (58%)	51.7–64
	Above my level	66 (27%)	51.7–64
	Way above my level	1 (0%)	0.1–2.3
	Unknown	9 (4%)	1.9–6.8
Height of fall	Bottom of wall	50 (20%)	15.8–25.9
	Middle of the wall	77 (31%)	15.8–25.9
	Top of the wall	116 (47%)	41.2–53.6
	Unknown	2 (2%)	0.2–3.0
Cause of fall			
Type of fall	Involuntary	208 (85%)	79.9–88.8
	Voluntary	35 (14%)	10.5–19.2
	Unknown	2 (1%)	0.2–2.9
Movement	Dynamic	100 (41%)	34.8–47.1
	Static	101 (41%)	35.2–47.5
	Unknown	9 (4%)	1.9–6.8
	Voluntary fall	35 (14%)	10.5–19.2
Cause of the fall	Foot slip	71 (29%)	23.7–35
	Dyno	54 (22%)	17.3–27.6
	Missed hold	51 (21%)	16.2–26.3
	Route finish	15 (6%)	3.7–9.9
	Balance loss	10 (4%)	2.2–7.3
	Hands let go of the hold	9 (4%)	1.9–6.8
	Downclimbing trouble	8 (3%)	1.7–6.3
	Tiredness	3 (1%)	0.4–3.5
	Stop wanted	2 (1%)	0.2–2.9
	Run and jump	2 (1%)	0.2–2.9
	Skate	1 (0%)	0.1–2.3
	Other	15 (6%)	3.7–9.9
	Unknown	4 (2%)	0.6–4.1
Fall kinematic			
Position at the start of the fall	Vertical	151 (62%)	55.4–67.5
	Leaning backward	64 (26%)	21–32
	Leaning forward	23 (9%)	6.3–13.7
	Unknown	7 (3%)	1.4–5.8
Rotation during the fall	Longitudinal rotation	77 (31%)	25.9–37.5
	Antero-posterior rotation	29 (12%)	25.9–37.5
	Back transverse rotation	17 (7%)	4.4–10.8
	Front transverse rotation	7 (3%)	1.4–5.8
	Multiple rotation	21 (9%)	5.7–12.7
	Without rotation	74 (30%)	24.8–36.2
	Unknown	20 (8%)	5.3–12.3
Position at reception	On feet and leaning	96 (39%)	33.3–45.4
	Standing on feet	84 (34%)	28.6–40.4
	Headfirst	29 (12%)	8.4–16.5
	Back or front to the pad	15 (6%)	3.7–9.9
	On buttocks	13 (5%)	3.1–8.9
	Unknown	8 (4%)	1.7–6.3
Landing surface	Pads	232 (94%)	91.1–96.9
	Between pads and wall	7 (3%)	1.4–5.8
	Between 2 pads	5 (2%	0.8–4.7
	Bump into someone	1 (1%)	0.1–2.3

### 3.2 Relationship between fall scenarios and injuries

An in-depth analysis was conducted on fall scenarios, examined from two perspectives: first, in relation to the most frequently affected body regions (upper and lower limbs) and second, based on the four main types of injury sustained (sprains, fractures, dislocations and tendon or ligament ruptures).

#### 3.2.1 Fall scenarios vs. injury locations

**Figure 2:**
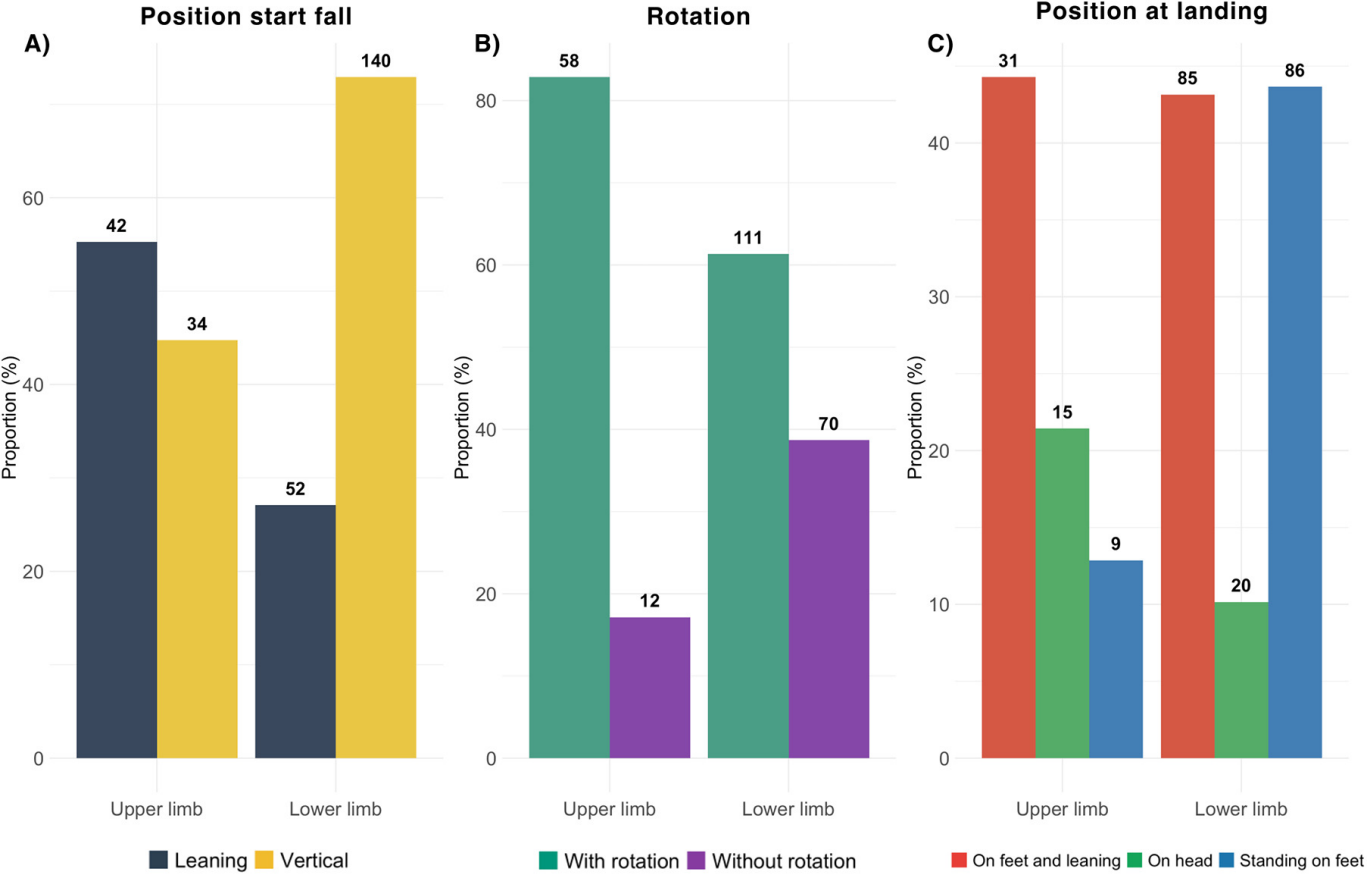
Description of fall kinematics according to location of injury for **(A)** the position at the start of the fall, **(B)** the rotation during the fall limited at with and without rotation **(C)** position at landing limited to the 3 most represented modalities.

Accidents leading to lower limb injuries were often characterized by an involuntary fall (84%) from a vertical wall 49% of the time (Table 4). The fall started in a vertical position (73%), often involved a rotation during the fall (61%) and a landing on the feet (87%), regardless of whether the climber was in a leaning or upright position (Figure 2). Lower limb injuries occurred at all fall heights, though falls from the top of the wall were slightly more frequent (42%).

**Table 4:** Descriptive presentation of fall scenarios according to the injury location.

Injury's location	Upper limbs		Lower limbs	
	*n* (%)	CI 95%	*n* (%)	CI 95%
Position start fall				
Vertical	34 (45%)	34.1–55.9	140 (73%)	66.2–78.7
Leaning backward	31 (41%)	30.4–52	37 (19%)	14.3–25.4
Leaning forward	11 (14%)	8.3–24.1	15 (8%)	4.8–12.5
Rotation				
Longitudinal	23 (33%)	23–44.5	66 (36%)	29.8–43.7
Antero-posterior	12 (17%)	10.1–27.6	17 (9%)	5.9–14.5
Back transverse	11 (16%)	9–26	8 (4%)	2.3–8.5
Front transverse	2 (3%)	0.8–9.8	5 (3%)	1.2–6.3
Multiple	10 (14%)	7.9–24.3	15 (8%)	5.1–13.2
Without	12 (17%)	10.1–27.6	70 (39%)	31.9–45.9
Position at reception				
Standing on feet	9 (13%)	6.9–22.7	86 (44%)	36.9–50.6
On feet and leaning	31 (44%)	33.2–55.9	85 (43%)	36.4–50.1
Head-first	15 (21%)	13.4–32.4	20 (10%)	6.7–15.2
Back or front to the pad	9 (13%)	6.9–22.7	2 (1%)	0.3–3.6
On the buttocks	6 (9%)	4–17.5	4 (2%)	0.8–5.1
Fall height				
Bottom of the wall	9 (12%)	6.5–21.5	49 (24%)	19–30.8
Middle of the wall	14 (19%)	11.6–29.3	67 (33%)	27.2–40.1
Top of the wall	51 (69%)	57.7–78.3	85 (42%)	35.7–49.2
Wall type				
Vertical	27 (36%)	26.4–47.9	96 (49%)	42.1–55.9
Steep	33 (45%)	33.8–55.9	50 (26%)	19.9–32
Slab	6 (8%)	3.8–16.6	25 (13%)	8.8–18.2
Corner	5 (7%)	2.9–14.9	9 (5%)	2.4–8.5
Roof/Overhang	3 (4%)	1.4–11.3	16 (8%)	5.1–12.8
Type of movement				
Dynamic	32 (44%)	33.5–55.9	84 (43%)	36.3–50.1
Static	32 (44%)	33.5–55.9	80 (41%)	34.4–48
Voluntary fall	8 (11%)	5.7–20.4	31 (16%)	11.4–21.7

Upper limb injuries mostly resulted from involuntary falls (88%) from the top (69%) of a steep wall (45%). Climbers were mostly in a leaning position at the beginning of the fall (55%), often rotated during the fall (83%), leading to a landing on their feet in a leaning position (57%).

The type of movement at the origin of the fall (static or dynamic) was similar for upper and lower limb injuries. However, a rotation during the fall more frequently occurred before upper limb injuries (Table 4).

#### 3.2.2 Fall scenarios vs. injury type

**Figure 3:**
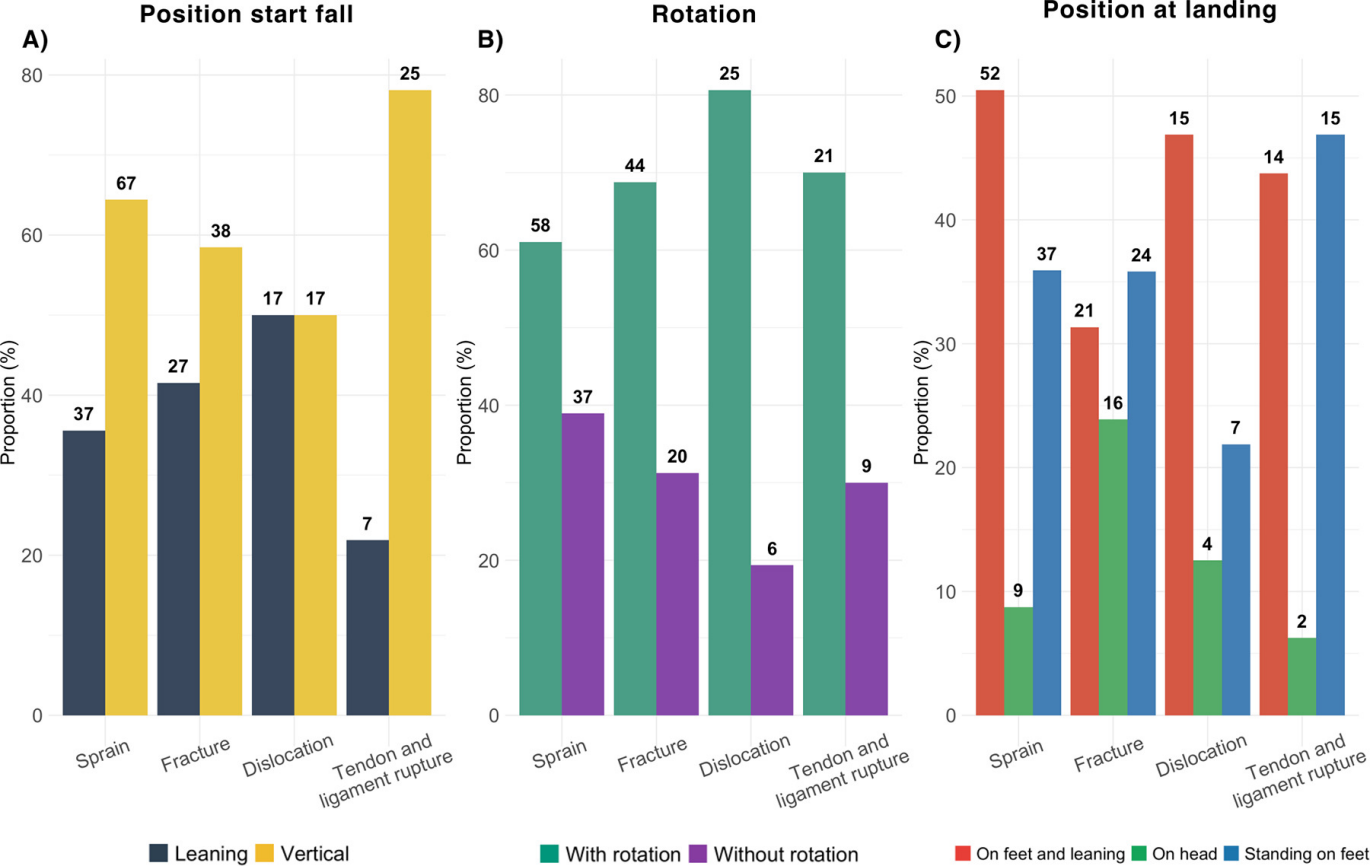
Description of fall kinematics according to type of injury for **(A)** the position at the start of the fall, **(B)** the rotation during the fall limited at with and without rotation **(C)** position at landing limited to the 3 most represented modalities.

Sprains mostly occurred following an involuntary fall (89%) from a vertical wall in 48% of the time (Table 5). The climber started the fall in a vertical position (64%), sustained a rotation during the fall (61%) and landed on the feet in a leaning position (50%) (Figure 3). Sprains had occurred at all fall heights. On the contrary, fractures were mostly caused by involuntary falls (87%) from the top (63%) of a vertical wall (42%), with the climber starting the fall in a vertical position (58%) and a rotation during the fall (69%). All landing types led to fractures though standing on feet was slightly more frequent. Dislocations mostly resulted from falls during a static movement (52%) from the top of the wall (70%) during which the climber sustained a rotation (81%) before landing on the feet while leaning (47%). Finally, tendon or ligament ruptures mostly occurred after a fall starting in a vertical position (78%) with a rotation during the fall (70%).

**Table 5:** Descriptive presentation of fall scenarios according to the injury type.

Injuries type	Sprain		Fracture		Dislocation		Tendon or ligament rupture	
	*n* (%)	CI 95%	*n* (%)	CI 95%	*n* (%)	CI 95%	*n* (%)	CI 95%
Position start fall								
Vertical	67 (64%)	54.9–73	38 (58%)	46.3–69.6	17 (50%)	34.1–65.9	25 (78%)	61.2–89
Leaning backward	29 (28%)	20.2–37.2	20 (31%)	20.9–42.8	12 (35%)	21.5–52.1	6 (19%)	8.9–35.3
Leaning forward	8 (8%)	3.9–14.4	7 (11%)	5.3–20.6	5 (15%)	6.4–30.1	1 (3%)	0.6–15.7
Rotation								
Longitudinal	30 (32%)	23.1–41.5	25 (39%)	28.1–51.3	8 (26%)	13.7–43.2	16 (53%)	36.1–69.8
Antero-posterior	14 (15%)	9–23.2	6 (9%)	4.4–19	4 (13%)	5.1–28.9	1 (3%)	0.6–16.7
Back transverse	5 (5%)	2.3–11.7	4 (6%)	2.5–15	5 (16%)	7.1–32.6	2 (7%)	1.8–21.3
Front transverse	3 (3%)	1.1–8.9	3 (5%)	1.6–12.9	0 (0%)	NA–NA	0 (0%)	NA–NA
Multiple	6 (6%)	2.9–13.1	6 (9%)	4.4–19	8 (26%)	13.7–43.2	2 (7%)	1.8–21.3
Without	37 (39%)	29.8–49	20 (31%)	21.2–43.4	6 (19%)	9.2–36.3	9 (30%)	16.7–47.9
Position at reception								
Standing on feet	37 (36%)	27.3–45.5	24 (36%)	25.4–47.8	7 (22%)	11–38.8	15 (47%)	30.9–63.6
On feet and leaning	52 (50%)	41–59.9	21 (31%)	21.5–43.2	15 (47%)	30.9–63.6	14 (44%)	28.2–60.7
Head-first	9 (9%)	4.7–15.8	16 (24%)	15.3–35.3	4 (12%)	5–28.1	2 (6%)	1.7–20.1
Back or front to the pad	2 (2%)	0.5–6.8	3 (4%)	1.5–12.4	4 (12%)	5–28.1	1 (3%)	0.6–15.7
On the buttocks	3 (3%)	1–8.2	3 (4%)	1.5–12.4	2 (6%)	1.7–20.1	0 (0%)	–
Fall Height								
Bottom of the wall	30 (28%)	20.4–37.2	7 (10%)	5.2–20	2 (6%)	1.7–19.6	9 (27%)	15.1–44.2
Middle of the wall	35 (33%)	24.6–42.1	18 (27%)	17.7–38.5	8 (24%)	12.8–41	11 (33%)	19.8–50.4
Top of the wall	42 (39%)	30.5–48.7	42 (63%)	50.7–73.3	23 (70%)	52.7–82.6	13 (39%)	24.7–56.3
Wall type								
Vertical	50 (48%)	38.7–57.6	28 (42%)	31.2–54.4	12 (38%)	22.9–54.7	15 (45%)	29.8–62
Steep	28 (27%)	19.3–36.2	20 (30%)	20.6–42.2	13 (41%)	25.5–57.7	13 (39%)	24.7–56.3
Slab	12 (12%)	6.7–19.1	7 (11%)	5.2–20.3	4 (12%)	5–28.1	2 (6%)	1.7–19.6
Corner	7 (7%)	3.3–13.2	4 (6%)	2.4–14.6	3 (9%)	3.2–24.2	0 (0%)	–
Roof/Overhang	7 (7%)	3.3–13.2	7 (11%)	5.2–20.3	0 (0%)	NA–NA	3 (9%)	3.1–23.6
Type of movement								
Dynamic	48 (47%)	37.3–56.2	30 (44%)	32.9–55.9	9 (29%)	16.1–46.6	16 (50%)	33.6–66.4
Static	43 (42%)	32.7–51.4	29 (43%)	31.6–54.5	16 (52%)	34.8–68	12 (38%)	22.9–54.7
Voluntary fall	12 (12%)	6.8–19.3	9 (13%)	7.1–23.3	6 (19%)	9.2–36.3	4 (12%)	5–28.1

### 3.3 Kinematics of the fall

**Figure 4:**
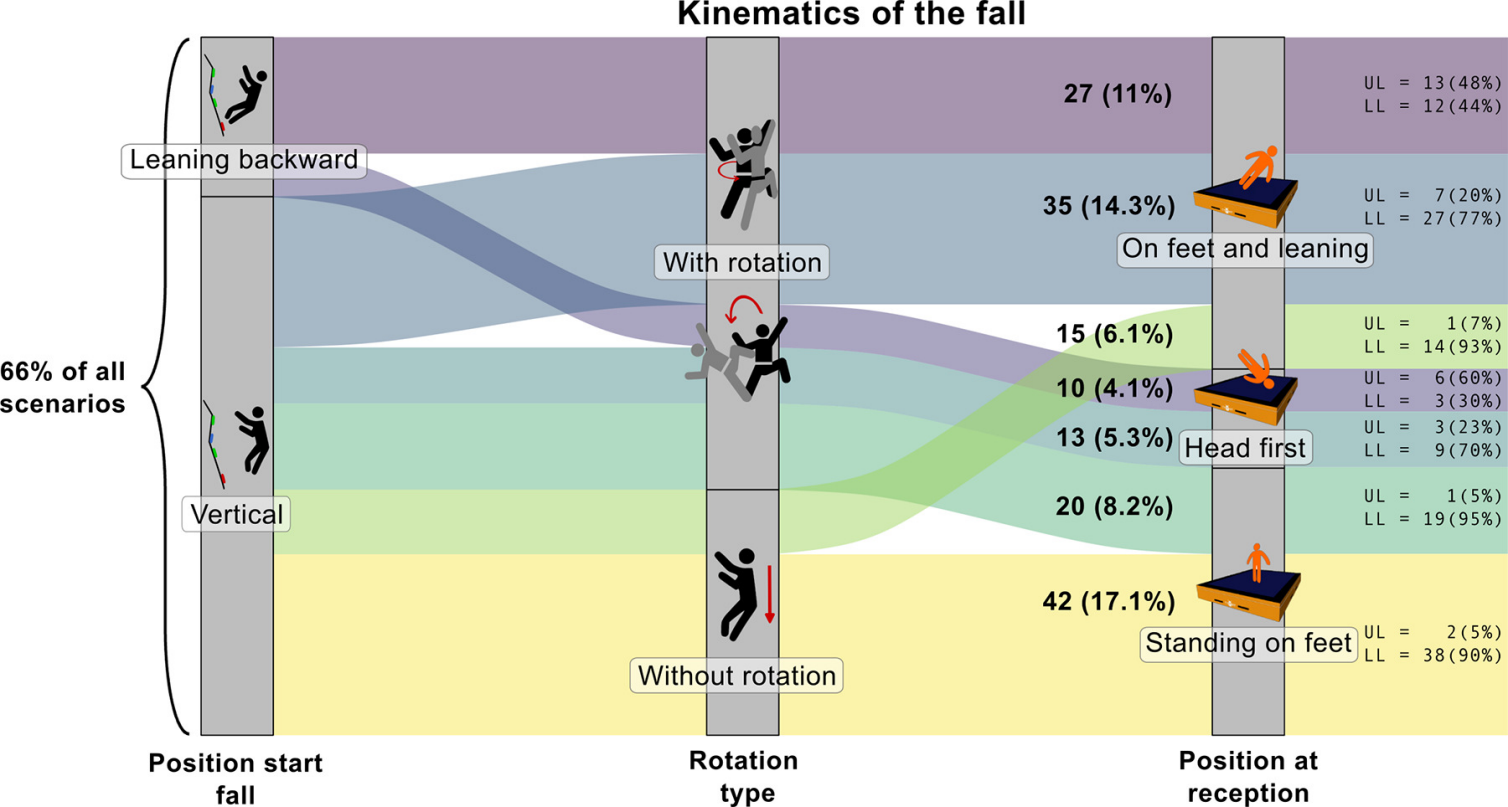
Description of the 7 most frequent accident scenarios, defined by the initial fall position, the rotation during the fall and the reception position. Counts and proportions are expressed as *n* (%) for each scenario. Injury locations associated with each scenario are indicated as UL for upper limb and LL for lower limb.

Figure 4 presents the seven most frequent accident kinematics, with five of them predominantly resulting in lower limb injuries. Kinematics were included in the graphic if they had at least 10 occurrences. The leaning position at the start of the fall refers exclusively to leaning backward, and rotation refers primarily to longitudinal rotation, due to the selection criteria explained above. Based on this, the three most frequent fall kinematics are:

- 1st kinematic (17% of all scenarios): The climber fell in a vertical position without a rotation, landed upright on the feet, and sustained a lower limb injury. Specifically, this scenario resulted in 13 ankle sprains, 7 knee tendon or ligament ruptures, 6 ankle fractures.
- 2nd kinematic (14% of all scenarios): The climber fell in a vertical position, rotates longitudinally during the fall, landed on the feet in a leaning position, and sustained a lower limb injury. Specifically, this scenario resulted in 10 ankle sprains, 5 knee tendon or ligament ruptures and 4 ankle fractures.
- 3rd kinematic (11% of all scenarios): The climber fell in a leaning position, rotated longitudinally during the fall, landed on the feet in a leaning position, and suffered of an upper limb injury. Specifically, this scenario resulted in 7 ankle sprains, 5 elbow dislocations, and 3 elbow sprains.

### 3.4 Factors influencing injury severity

**Figure 5:**
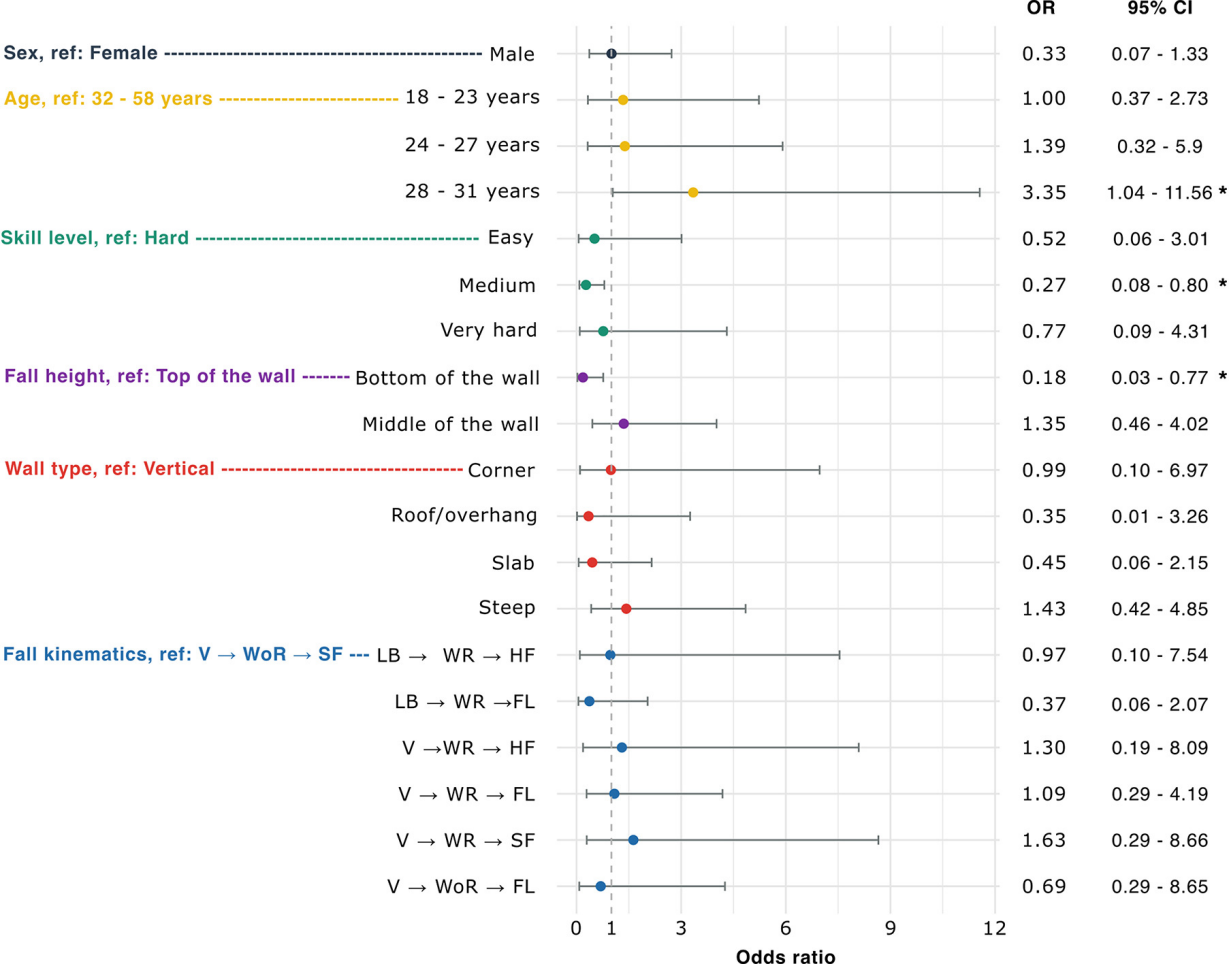
Multiple logistic regression assessing the effect of fall scenario parameters on injury severity [“minor injury” (UIAA 1 and 2) vs. “severe injury” (UIAA 3 and 4)]. In the figure, dots represent the odds ratios (OR), and horizontal lines indicate the 95% confidence intervals (95% CI). Reference categories for each variable are indicated on the left. Significant results are marked with stars, where * denotes *p* ≤ 0.05. Type of movement for the fall kinematics variable are indicate as V for vertical, LB for leaning backward, WR for with rotation, WoR for without rotation, HF for head first, FL for on feet and leaning and SF for standing on feet.

Figure 5 illustrates the results of the logistic regression model, presenting odds ratios and confidence intervals for assessing the risk of serious injury. Climbers aged 28–31 were more likely to sustain a severe injury (OR: 3.35, 95% CI: 1.04–11.56) than those aged 32–58 (Figure 5). In contrast, participants who rated their skill level as medium (6a–6c+) were at a lower risk of severe injury (OR: 0.27, 95% CI: 0.08–0.80) than those who rated it as hard (7a–7b+). Additionally, falls from the bottom of the wall were associated with a lower risk of severe injury (OR: 0.18, 95% CI: 0.03–0.77) than falls from the top section. Sex, wall type, and fall kinematics were not found to be significantly associated with the risk of severe injury.

## 4 Discussion

Fall-related injuries are almost always involuntary, suggesting that the risk of injury is lower when a fall is intentional. This can be attributed to the climber's greater awareness at the moment of jumping off the wall, resulting in a more controlled landing on the pad. This control allows the body to maintain better stability on impact and achieve better muscular activation, especially in the lower limbs, resulting in more effective absorption of the energy generated by the impact through bending the legs. In voluntary falls, any rotational motion is self-initiated and controlled, potentially reducing injury risk. In addition, voluntary falls were more likely to occur after a partial descent of the route and, therefore, more frequently from the bottom of the wall. This was associated with a risk of severe injury that was almost 82% lower than falling from the top section of the wall (Figure 5). In fact, falling from the top of the wall induces higher impact speed on the pads, resulting in higher forces on the body, which are known to increase the risk of injury. This may also explain why most injuries occurred during falls from the middle and top section of the wall. Additionally, vertical and steep walls tend to be the most prone to accidents (Figure 5), but they are also the most frequent type of wall in bouldering gyms.

Two main types of fall kinematics emerged from the study. The first one regroups the first and second kinematics observed (Figure 4). and was mostly at risk of sprains, tendon/ligament ruptures and fractures. These injuries may be induced by the sinking of the feet and the ankles in the pad during the impact, favoring high ankle motion (e.g., supination) leading to the injury (9, 29), potentially combined with a rotation of the rest of the body. A possible explanation for the occurrence of sprains versus fractures for the same scenario could be the fall height, as observed in climbing study (8). A greater fall height increases impact speed, and the forces exerted on the lower limbs during landing, and therefore the likelihood of fracture. Indeed, more fractures are observed when climbers fall from the top of the wall, while this effect is less pronounced for sprains and tendon or ligament ruptures (Table 5). This fall kinematic is very similar to the one of voluntary falls. This raises the question of whether its high occurrence among injurious falls is due to its overall prevalence among all falls, both injurious and non-injurious, or if it is inherently more dangerous. Therefore, investigating non-injurious falls in a bouldering gym could help determine the prevalence of risky kinematics.

The second main type of fall kinematic (third kinematic described on Figure 4) involve climbers mostly leaning backwards (rather than frontwards) at the start of the fall (Table 3) which might be linked to the high occurrence of the upper limb injuries after a fall from a steep wall (Table 4). This leaning start might generate a rotation during descent, leading to an unstable landing position upon impact with the pads. The climber might therefore instinctively use the upper limbs to absorb the impact with the pads, leading to the injury. This position could explain the number of injuries observed in the elbow region following this scenario.

Finally, none of the observed kinematics were found to be significantly associated with a higher risk of severe injury (Figure 5). This lack of association may be attributed to the limited sample size in the fall kinematics groups, which ranged from 10 to 42 observations.

Previous findings were observed in a predominantly female population (62%), contrasting with other bouldering studies where males were predominant (15) or genders were equally represented (22). This gender imbalance may be due to females participating more frequently in sport-related surveys (30). The demographics of this study, including average age (16, 22), number of bouldering sessions per week (15), and skill level (16, 22), are consistent with previous studies on similar populations. Climbers aged under 32 years tend to sustain more severe injuries, particularly those aged 28–31, who are 3.3 times more likely to be severely injured (Figure 5). Conversely, having a medium skill level (6a–6c+) reduces the likelihood of severe injury by 73%. These findings contrast with previous literature (31). The observation that 27% of respondents had sustained previous injuries differs from another study, which reported a majority (59%) of respondents with previous injuries (15). This discrepancy may be attributed to differences in injury definitions, as the other study consider all bouldering injuries (acute as overuse). Additionally, 135 participants were excluded due to a high number of missing answers (NAs). Most of them did not respond to 80% or 90% of the questions, suggesting they may not have begun the survey. The information and consent section, appearing as the second page, may have contributed to this high dropout rate.

The injury mechanism (e.g., falling, bouldering) directly impacts the localization, type, and severity of injuries reported in studies (8, 9, 15–20, 22, 32). In this study, the lower limbs were the most affected body region, accounting for nearly 70% of all reported injuries. This high prevalence can be attributed to the study's focus on fall-related injuries, which have been closely associated with lower limb injuries (9, 15, 16, 22). In contrast, studies that included other injury mechanisms, such as those inherent to bouldering itself, have found many overuse injuries primarily affecting the upper limbs, with the hand, fingers, and thumbs being the most affected (15, 16). Regarding injury typology, sprains, fractures, and dislocations were the most commonly reported injuries, and especially ankle sprains emerged as the predominant injury type, reinforcing previous findings with similar injury mechanisms (9, 22). Additionally, dislocations predominantly affected the upper limb, with 85% of cases involving this body part, a pattern consistent with injury distribution previously reported (9). Furthermore, 64% of the injuries reported in this study were classified as UIAA 2% and 20% as UIAA 3+, further supporting previous findings with similar injury mechanisms (9, 15). These results highlight the vulnerability of the lower limbs, particularly the ankle, as well as the notable occurrence of dislocations affecting the upper limbs after a fall in bouldering.

To reduce lower limb injuries, a study suggests minimizing excessive indentation of the pads by the feet (29). Increasing pad rigidity could prevent excessive ankle supination, thereby reducing force on ligament structures and lowering injury risk. However, this solution may be ineffective or even worse for high-impact energy scenarios. Consequently, further analysis is essential to ascertain the optimal balance between pad rigidity and the force transmitted to the climber upon impact. Additionally, advising coaches to conduct training sessions on voluntary falls and teaching athletes to fall with their arms crossed in front and rolling onto their backs could help to reduce the observed upper limb injuries.

Finally, this study showed the most common fall scenarios that should be cushioned by the pads provided in bouldering gyms. Nevertheless, these findings represent only a partial contribution to the future safety pads improvement and should be completed by a more detailed investigation of fall kinematics and impact conditions, potentially through video analysis. Further research could also involve reconstructing complete fall scenarios using finite element numerical modelling to provide deeper insights into injury mechanisms and assess the influence of different pad designs on injury outcomes.

This study has several limitations that should be considered when interpreting the findings. First, as a retrospective study, the data were collected through self-reported surveys completed by climbers themselves. This approach could introduce inaccuracies in the description of the injury, including its location, type, and severity classification. Second, the accidents occurred in various unidentified bouldering gyms. Consequently, it is impossible to obtain information about the pads used in these gyms at the time of the accident, such as their rigidity, thickness or wear, or regarding the route opening. This limits our ability to draw conclusions about environmental or facility-related contributors to injuries. Third, the survey did not specify a time limit for the reported injuries. Consequently, older injuries might be described with less accuracy due to the climber's diminished memory of the event and the specifics of the injury. This potential recall bias could affect the reliability of the data regarding the circumstances, mechanism and nature of the injuries. Fourth, due to the lack of literature on the effect size between the predictor variables (fall scenarios) and the response variable, a G*Power analysis could not be performed, as it would yield arbitrary estimations. It should be noted that a slight change in the expected effect size from 1.3 to 1.5, for example, can halve the required sample size, underscoring the sensitivity of such calculations. Power analyses are generally uncommon in exploratory epidemiological injury studies, which often do not test specific hypotheses. Consequently, our findings may be less robust due to this uncertainty. However, this study provides valuable preliminary data for estimating effect sizes in future research. Finally, the sample size in this study is relatively small due to the strict inclusion criteria. This can result in limited representation within the response groups, particularly for variables with numerous possible modalities.

In conclusion, this study details fall scenarios linked to bouldering injuries. The most accident-prone scenario involves falling from the upper part of a vertical or steep wall, maintaining a vertical position without rotation, and landing on the feet, often resulting in lower limb injuries like ankle sprains. Two distinct scenarios for lower and upper limb injuries highlight the impact of injury mechanisms. These findings should guide the development of future preventive measures and protective equipment, such as pads, to improve bouldering safety.

## Data Availability

The datasets presented in this article are not readily available because the dataset must remain confidential for the moment. Requests to access the datasets should be directed to erwan.beurienne@univ-eiffel.fr.
