# Supplementary material for: The art of falling: identifying the falls scenarios associated with bouldering injuries
Source: Front Sports Act Living. 2025 Jul 17;7:1609133. doi: 10.3389/fspor.2025.1609133 (PMC12312403; doi:10.3389/fspor.2025.1609133)
Supplement: Supplementary file 1 [file Datasheet1.pdf]

Accidentologie en escalade de bloc

Si vous avez été victime d'une blessure (i.e. toute plainte ou manifestation de douleur musculo-squelettique ayant conduit à un changement dans les modes, les durées, les intensités ou les fréquences habituelles des entraînements ou des compétitions) résultant d'une chute en salle d'escalade de bloc intérieure et que vous avez 18 ans ou plus, votre participation nous aidera grandement à diminuer le risque de blessure des grimpeur•euses.

Contexte :

Depuis plusieurs années, la pratique de l'escalade est en plein essor. Ceci se traduit par une forte augmentation du nombre de pratiquants et de salles d'escalade en France. Parallèlement à ce gain de popularité, le nombre de blessures recensées dans les différentes sous-disciplines de l'escalade est en augmentation de près de 50% sur la période 2008-2016. La sous-discipline la plus touchée par

cette augmentation est le bloc en intérieur. Les blessures en bloc affectent majoritairement le membre inférieur et sont causées par des chutes (volontaires ou involontaires).

Le présent questionnaire a pour objectif de définir les scénarios de chute les plus fréquents chez les grimpeur•euses de bloc ainsi que de construire une cartographie des blessures résultant de ces chutes.

Il y a 47 questions dans ce questionnaire.

Quel est votre âge ?

❗ Seuls des nombres peuvent être entrés dans ce champ.
Veuillez écrire votre réponse ici :

Ans

Quel est votre sexe ?

Veuillez sélectionner une seule des propositions suivantes :

- ◯ Féminin
- ◯ Masculin

Quelle est votre taille ? (en cm)

❗ Seuls des nombres peuvent être entrés dans ce champ.
Veuillez écrire votre réponse ici :

[ ]

cm

Quel est votre poids ? (en kg)

❗ Seuls des nombres peuvent être entrés dans ce champ.
Veuillez écrire votre réponse ici :

[ ]

kg

Parmi les méthodes d’apprentissage de l’escalade énumérées ci-dessous, sélectionnez celle·s qui vous a ou ont permis de débuter votre pratique.

❗ Cochez la ou les réponses
Veuillez choisir toutes les réponses qui conviennent :

- ☐ En club
- ☐ A l'école
- ☐ Avec des ami·es
- ☐ En famille
- ☐ Seul·e
- ☐ Autre: [ ]

Classer les éléments de la liste ci-dessous, de la pratique la plus réalisée (en haut) à la moins réalisée (en bas).
Les disciplines que vous n’auriez jamais pratiquées ne sont pas à classer.

❶ Vos réponses doivent être différentes, et vous devez les classer dans l’ordre.
Numérotez chaque case dans l’ordre de vos préférences de 1 à 7

[ ] En tête/moulinette - En salle

[ ] En tête/moulinette - En extérieur

[ ] Bloc - En salle

[ ] Bloc - En extérieur

[ ] Alpinisme

[ ] Traditionnelle (terrain d'aventures)

[ ] Autre

Par semaine, combien d'heures (en moyenne) de pratique d'escalade effectuez-vous ?

❶ Seuls des nombres peuvent être entrés dans ce champ.
Veuillez écrire votre réponse ici :

[ ]

heures par semaine

Au moment de votre **accident le plus grave**, depuis combien de temps pratiquiez-vous régulièrement l'escalade de bloc ?

Veuillez écrire votre réponse ici :

an•s

Au moment de votre **accident le plus grave**, quel était votre **niveau régulier** à vue (réussite du premier coup) ? Puis après travail (réussite après plusieurs tentatives)

Correspondance des niveaux de difficulté			
Cotation de Bleau	Climb'Up	Arkose	Réponses proposées
1 - 5+			Facile
6A - 6C+			Intermédiaire
7A - 7B+			Difficile
7C et supérieur			Très difficile

Choisissez la réponse appropriée pour chaque élément :

	Facile	Intermédiaire	Difficile	Très difficile	Je ne sais pas
**Niveau régulier à vue :**	◯	◯	◯	◯	◯
**Niveau régulier après travail :**	◯	◯	◯	◯	◯

Au moment de votre **accident le plus grave,** combien de fois et combien d'heure par semaine pratiquiez vous l'escalade **tout type confondus** (temps de pratique incluant l'assurage / le repos au pied des voies ou des blocs) ?

**Nombre de séance•s par semaine :**	
**Nombre d'heure•s par semaine :**	

Au moment de votre **accident le plus grave**, combien de fois et combien d'heure par semaine pratiquiez-vous **l'escalade de bloc spécifiquement** ?

**Nombre de séance•s par semaine :**	
**Nombre d'heure de pratique par semaine :**	

Quelle était la date de votre accident ?

Veuillez entrer une date :

Concernant votre état de fatigue, comment vous sentiez vous juste avant votre accident ?

Choisissez la réponse appropriée pour chaque élément :

Très fatigué•e	Fatigué•e	Ni l'un, ni l'autre	Pas fatigué•e	Pas du tout fatigué•e
◯	◯	◯	◯	◯

À quelle hauteur du mur étiez-vous au moment de votre accident ?

Veuillez sélectionner une seule des propositions suivantes :

- ◯ Bas du mur
- ◯ Milieu du mur
- ◯ Haut du mur
- ◯ Je ne sais pas

Quel était le type de mur que vous grimpiez au moment de votre accident ?

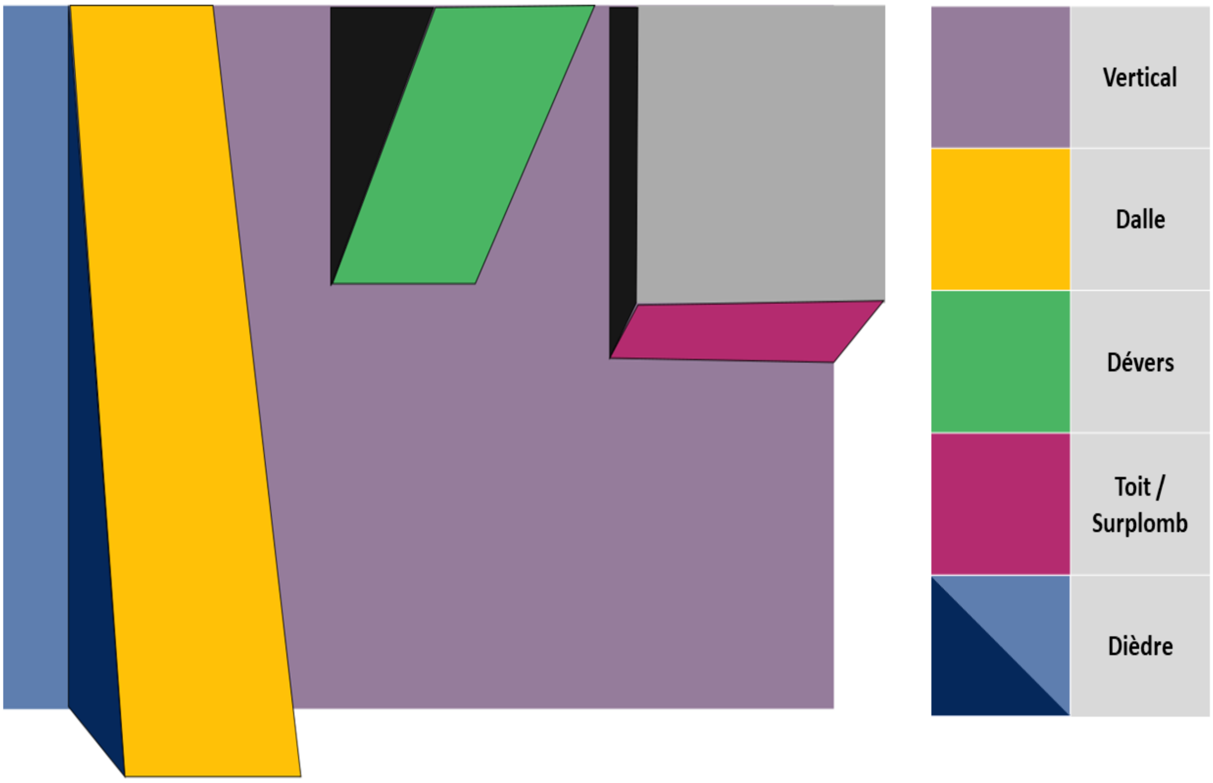

Veuillez sélectionner une seule des propositions suivantes :

- ◯ Vertical
- ◯ Dalle
- ◯ Dévers
- ◯ Toit / Surplomb
- ◯ Dièdre
- ◯ Je ne sais pas

Au moment de l'accident, vous effectuiez une tentative à vue (réussite après une tentative) ou après travail du bloc (réussite après plusieurs tentatives) ?

Veuillez sélectionner une seule des propositions suivantes :

- ◯ Tentative à vue
- ◯ Tentative après travail

Par rapport à votre niveau habituel à vue, au moment de l'accident, comment situez-vous le bloc que vous tentiez en termes de difficulté ?

Choisissez la réponse appropriée pour chaque élément :

Très en-dessous de mon niveau	En-dessous de mon niveau	Juste à mon niveau	Au-dessus de mon niveau	Très au-dessus de mon niveau
◯	◯	◯	◯	◯

Par rapport à votre niveau habituel après travail, au moment de l'accident, comment situez-vous le bloc que vous tentiez en termes de difficulté ?

Choisissez la réponse appropriée pour chaque élément :

Très en-dessous de mon niveau	En-dessous de mon niveau	Juste à mon niveau	Au-dessus de mon niveau	Très au-dessus de mon niveau
◯	◯	◯	◯	◯

Pendant votre chute, avez-vous percuté un obstacle ?

Veuillez choisir toutes les réponses qui conviennent :

- ☐ Volume·s
- ☐ Prise·s
- ☐ Un ou une autre grimpeur·euse
- ☐ Aucun obstacle percuté
- ☐ Autre: ____________

Où avez-vous atterri après votre chute ?

Veuillez choisir toutes les réponses qui conviennent :

- ☐ Sur les matelas
- ☐ Entre deux matelas
- ☐ Entre un matelas et un mur
- ☐ Sur quelqu'un
- ☐ Sur un objet posé sur les matelas
- ☐ Hors des matelas
- ☐ Je ne sais pas
- ☐ Autre: ____________

Votre chute était-elle volontaire ou involontaire ?

Veuillez sélectionner une seule des propositions suivantes :

- ◯ Volontaire (saut sur le matelas)
- ◯ Involontaire (chute non voulue ou non préméditée)

Pourquoi avez-vous pris la décision de sauter du mur ?

Veuillez sélectionner une seule des propositions suivantes :

- ◯ J’étais fatigué
- ◯ J’avais fini le bloc et j’ai donc sauté
- ◯ J’avais des difficultés à désescalader
- ◯ Je souhaitais m’arrêter là
- ◯ Autre ____________

Au moment de votre chute, étiez-vous en train de réaliser un mouvement statique ou dynamique ?

Veuillez sélectionner une seule des propositions suivantes :

- ◯ Mouvement statique (Mouvement lent et contrôlé où il est possible de revenir en arrière)
- ◯ Mouvement dynamique (Mouvement rapide où il n'est pas possible de revenir en arrière)
- ◯ Je ne sais pas

Quel a été l'évènement à l'origine de votre chute ?

Veuillez sélectionner une seule des propositions suivantes :

- ◯ Prise mal attrapée (et donc la main glisse)
- ◯ Prise défectueuse (qui tourne, casse…)
- ◯ Zipette (glissade d'un pied d'appui sur une prise)
- ◯ Plus de force dans les bras, les mains ont lâché la prise
- ◯ Perte d'équilibre
- ◯ Autre ____________

Quel a été l'évènement à l'origine de votre chute ?

Veuillez sélectionner une seule des propositions suivantes :

- ◯ Jeté (mouvement d'impulsion en vue d'atteindre une prise qui n'aurait pas pu être atteinte normalement)
- ◯ Run and Jump (course du sol vers la paroi, permettant d'attraper une prise ou un volume non atteignable)
- ◯ Skate (course latérale sur des volumes avant d'atteindre les prises)
- ◯ Zipette (glissade d'un pied d'appui sur une prise)
- ◯ Prise mal attrapée (et donc la main glisse)
- ◯ Prise défectueuse (qui tourne, casse…)
- ◯ Autre

Quel a été l'évènement à l'origine de votre chute ?

Veuillez sélectionner une seule des propositions suivantes :

- ◯ Jeté (mouvement d'impulsion en vue d'atteindre une prise qui n'aurait pas pu être atteinte normalement)
- ◯ Run and Jump (course du sol vers la paroi, permettant d'attraper une prise ou un volume non atteignable)
- ◯ Skate (course latérale sur des volumes avant d'atteindre les prises)
- ◯ Zipette (glissade d'un pied d'appui sur une prise)
- ◯ Prise mal attrapée (et donc la main glisse)
- ◯ Prise défectueuse (qui tourne, casse…)
- ◯ Plus de force dans les bras, les mains ont lâché la prise
- ◯ Perte d'équilibre
- ◯ Autre

Indiquez le nombre de blessures (i.e. toute plainte ou manifestation de douleur musculo-squelettique ayant conduit à un changement dans les modes, les durées, les intensités ou les fréquences habituelles des entraînements ou des compétitions) suite à votre accident ?

Veuillez sélectionner une seule des propositions suivantes :

- ◯ 1
- ◯ 2
- ◯ 3
- ◯ 4
- ◯ 5 et +

Sur quelle partie du corps se situait votre blessure ?

Groupe principaux	Catégorie	Equivalent OSICS	Légende
Tête et cou	Tête / Face	H	
	Cou / Vertèbre cervicale	N	
Membre supérieur	Epaule / Clavicule	S	
	Bras (partie proximale)	U	
	Coude	E	
	Avant-bras (partie distale)	R	
	Poignet	W	
	Main / Doigts / Pouce	P	
Tronc	Sternum / Côtes / Haut du dos	C,D	
	Abdomen (ventre)	O	
	Bas du dos / Pelvis / Sacrum	B,L	
Membre inférieur	Hanche / Aine	G	
	Cuisse	T	
	Genou	K	
	Jambe / Tendon d'achille	Q,A	
	Cheville	A	
	Pied / Orteils	F	

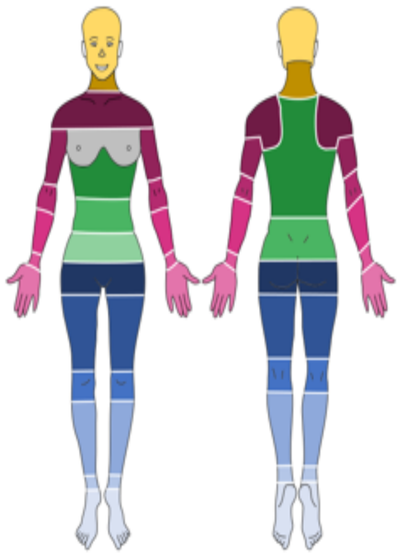

Veuillez sélectionner une seule des propositions suivantes :

- ◯ (Tête et cou) Face
- ◯ (Tête et cou) Tête
- ◯ (Tête et cou) Cou / Vertèbre cervicale
- ◯ (Membre supérieur) Epaule / Clavicule

- ◯ (Membre supérieur) Bras (partie proximale)
- ◯ (Membre supérieur) Coude
- ◯ (Membre supérieur) Avant-bras (partie distale)
- ◯ (Membre supérieur) Poignet
- ◯ (Membre supérieur) Main / Doigts / Pouce
- ◯ (Tronc) Sternum / Côtes / Haut du dos
- ◯ (Tronc) Abdomen (ventre)
- ◯ (Tronc) Bas du dos / Pelvis / Sacrum
- ◯ (Membre inférieur) Hanche / Aine
- ◯ (Membre inférieur) Cuisse
- ◯ (Membre inférieur) Genou
- ◯ (Membre inférieur) Jambe / Tendon d'achille
- ◯ (Membre inférieur) Cheville
- ◯ (Membre inférieur) Pied / Orteils

Quelle était la nature de votre blessure ?

Veuillez sélectionner une seule des propositions suivantes :

- ◯ Fracture
- ◯ Entorse
- ◯ Luxation
- ◯ Ecchymoses (bleus)
- ◯ Blessure cutanée
- ◯ Traumatisme crânien sans perte de connaissance
- ◯ Traumatisme crânien avec perte de connaissance
- ◯ Rupture de tendon
- ◯ Tendinite
- ◯ Je ne sais pas
- ◯ Autre

Quelle était la sévérité de votre blessure ?

Veuillez sélectionner une seule des propositions suivantes :

- ◯ Blessure légère (exemple : ecchymoses, blessure cutanée, foulures, tendinite)
- ◯ Blessure moyennement grave, intervention médicale et non chirurgicale (exemple : fractures non déplacées, ruptures de tendons, luxations, entorse)
- ◯ Blessure grave, intervention chirurgicale nécessaire (exemple : luxation grave, fractures ouvertes)
- ◯ Danger mortel aïgu, polytraumatisme, dommages permanents
- ◯ Je ne sais pas

Sur quelles parties du corps se situaient vos blessures ?

Si vous avez subi plusieurs blessures, merci d'indiquer seulement les 2 plus importantes (i.e. celles ayant entraîné le plus d'immobilisation ou de modifications dans les habitudes « d'entraînement »)

Groupe principaux	Catégorie	Equivalent OSICS	Légende
Tête et cou	Tête / Face	H	
	Cou / Vertèbre cervicale	N	
Membre supérieur	Epaule / Clavicule	S	
	Bras (partie proximale)	U	
	Coude	E	
	Avant-bras (partie distale)	R	
	Poignet	W	
	Main / Doigts / Pouce	P	
Tronc	Sternum / Côtes / Haut du dos	C,D	
	Abdomen (ventre)	O	
	Bas du dos / Pelvis / Sacrum	B,L	
Membre inférieur	Hanche / Aine	G	
	Cuisse	T	
	Genou	K	
	Jambe / Tendon d'achille	Q,A	
	Cheville	A	
	Pied / Orteils	F	

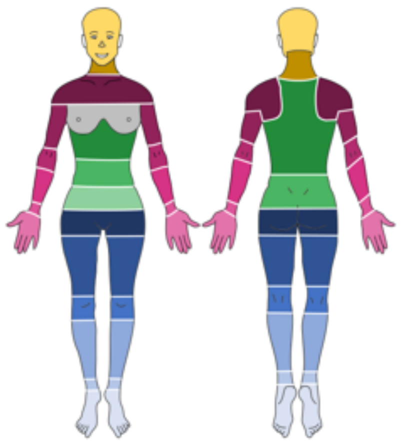

Choisissez la réponse appropriée pour chaque élément :

	Blessure n°1	Blessure n°2
**Face**	○	○
**Tête**	○	○
**Cou / Vertèbre cervicale**	○	○
**Epaule /Clavicule**	○	○
**Bras (partie proximale)**	○	○
**Coude**	○	○
**Avant-bras (partie distale)**	○	○
**Poignet**	○	○
**Main / Doigts / Pouce**	○	○
**Sternum / Côtes / Haut du dos**	○	○

**Abdomen (ventre)**	○	○
**Bas du dos / Pelvis / Sacrum**	○	○
**Hanche / Aine**	○	○
**Cuisse**	○	○
**Genou**	○	○
**Jambe / Tendon d'achille**	○	○
**Cheville**	○	○
**Pied / Orteils**	○	○

Quelles étaient les natures de vos blessures ?

Si vous avez subi plusieurs blessures, merci d'indiquer seulement les 2 plus importantes (i.e. celles ayant entraîné le plus d'immobilisation ou de modifications dans les habitudes « d'entraînement »).

Choisissez la réponse appropriée pour chaque élément :

	**Blessure n°1**	**Blessure n°2**
**Fracture**	◯	◯
**Entorse**	◯	◯
**Luxation**	◯	◯
**Ecchymoses (bleus)**	◯	◯
**Blessure cutanée**	◯	◯
**Traumatisme crânien sans perte de connaissance**	◯	◯
**Traumatisme crânien avec perte de connaissance**	◯	◯
**Rupture de tendon**	◯	◯
**Tendinite**	◯	◯
**Autre**	◯	◯
**Je ne sais pas**	◯	◯

Indiquez ici la première blessure que vous avez subie

Veuillez écrire votre réponse ici :

Indiquez ici la seconde blessure que vous avez subie

Veuillez écrire votre réponse ici :

Quelles étaient la sévérité de vos blessures ?

Si vous avez subi plusieurs blessures, merci d'indiquer seulement les 2 plus importantes (i.e. celles ayant entraîné le plus d'immobilisation ou de modifications dans les habitudes « d'entraînement »)

Choisissez la réponse appropriée pour chaque élément :

	**Blessure n°1**	**Blessure n°2**
**Blessure légère (exemple : ecchymoses, blessure cutanée, foulures, tendinite)**	○	○
**Blessure moyennement grave, intervention médicale et non chirurgicale (exemple : fractures non déplacées, ruptures de tendons, luxations, entorse)**	○	○
**Blessure grave, intervention chirurgicale nécessaire (exemple : luxation grave, fractures ouvertes)**	○	○
**Danger mortel aïgu, polytraumatisme, dommages permanents**	○	○
**Je ne sais pas**	○	○

Au moment ou vous remplissez ce questionnaire, êtes vous encore blessé ?

Veuillez sélectionner une seule des propositions suivantes :

- ◯ Oui
- ◯ Non

Combien de jours se sont écoulés entre le moment de votre accident et le jour où vous avez pu reprendre pleinement votre entraînement ?

Veuillez écrire votre réponse ici :

[ ]

Jour•s

Parmi les schémas ci-dessous (représentant un grimpeur vu de profil), lequel est le plus représentatif de votre position au début de votre chute ?

Veuillez cliquer directement sur l'image pour la sélectionner

Veuillez sélectionner une seule des propositions suivantes :

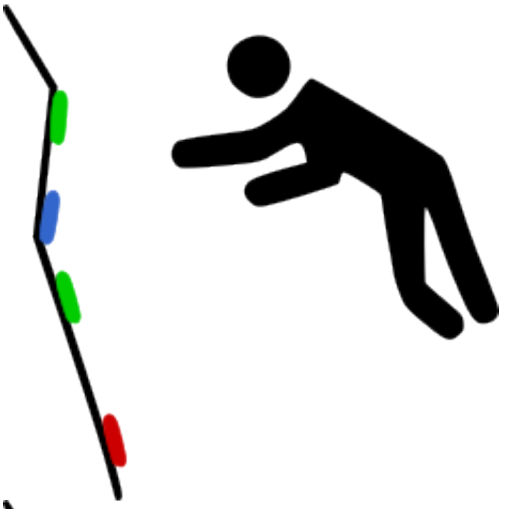

Penché vers l'avant

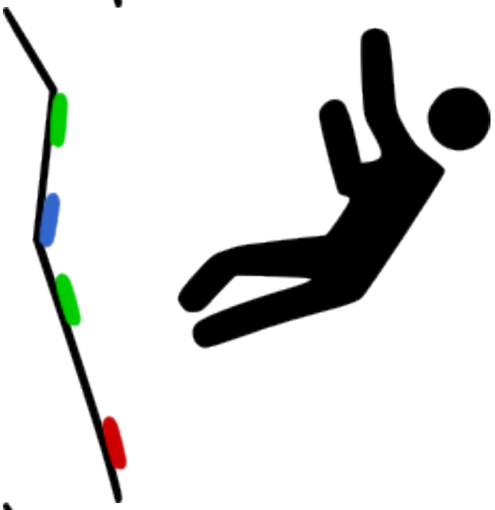

Penché vers l'arrière

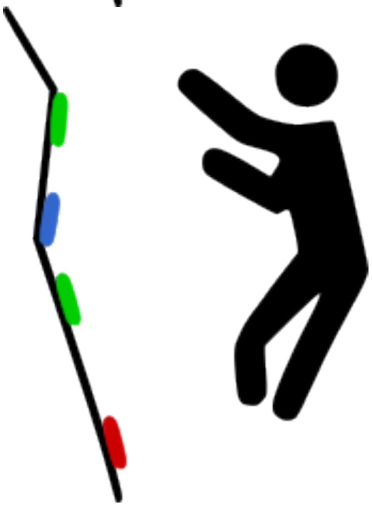

À la verticale

Je ne sais pas

Parmi les schémas ci-dessous (représentant le grimpeur de dos), lequel est le plus représentatif de votre position au début de votre chute ?

La droite et la gauche ne sont pas différenciées

Veuillez cliquer directement sur l'image pour la sélectionner

Veuillez sélectionner une seule des propositions suivantes :

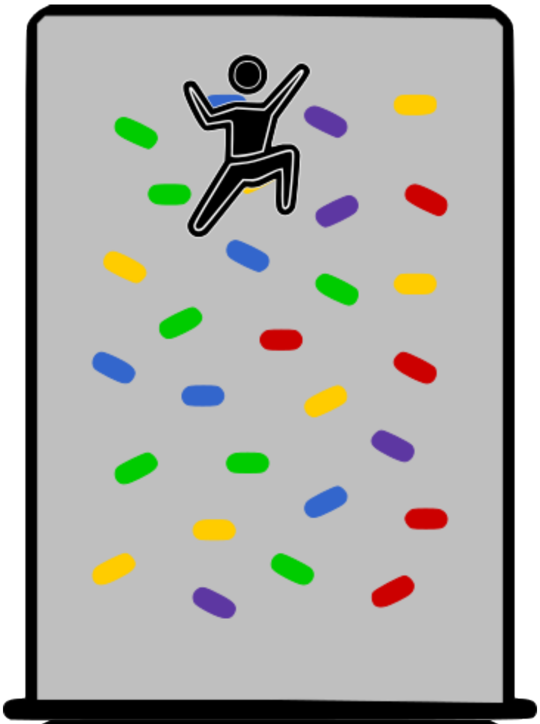

○ La tête en haut

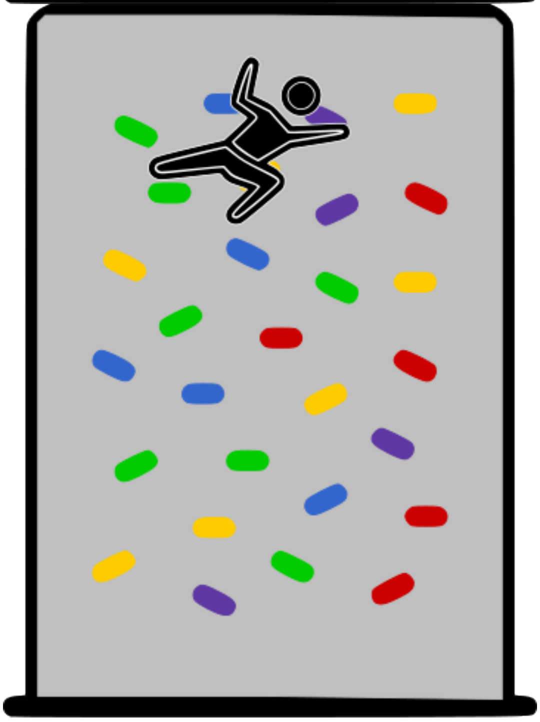

○ Couché à 45° sur le coté

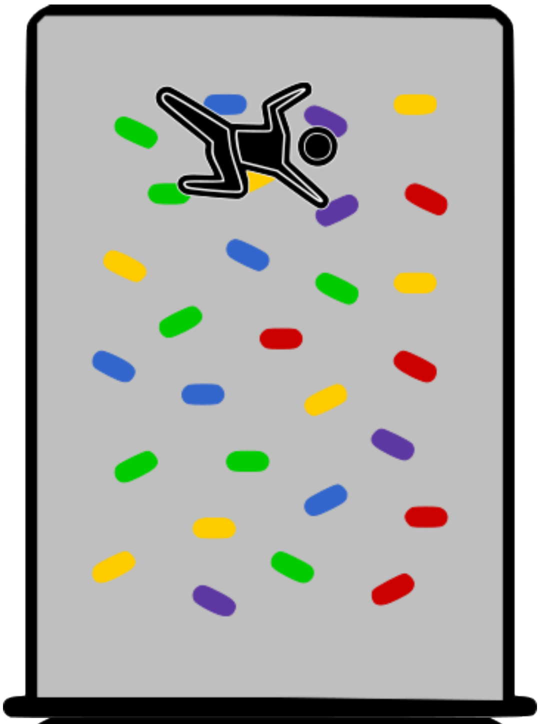

○ Couché à 90° sur le coté

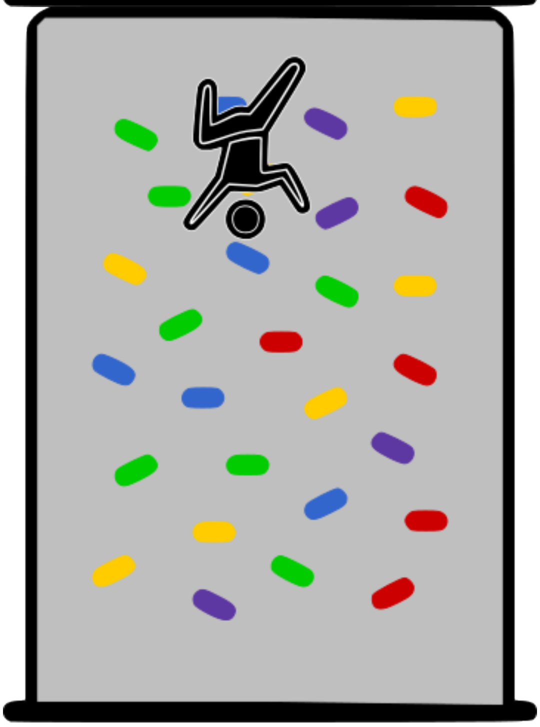

○ La tête en bas

○ Je ne sais pas

Etiez-vous en rotation pendant la chute ?

Si oui, la ou lesquelles de ces rotations représente·nt le mieux votre chute ?

Veuillez cliquer directement sur l'image pour la sélectionner

Veuillez choisir toutes les réponses qui conviennent :

☐ Oui

☐ Non

☐ 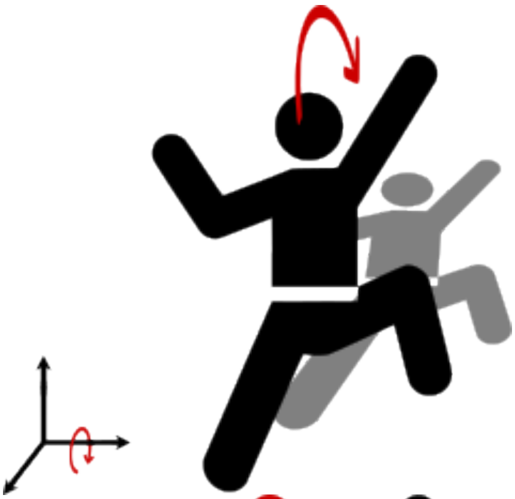

Rotation vers l'avant

☐ 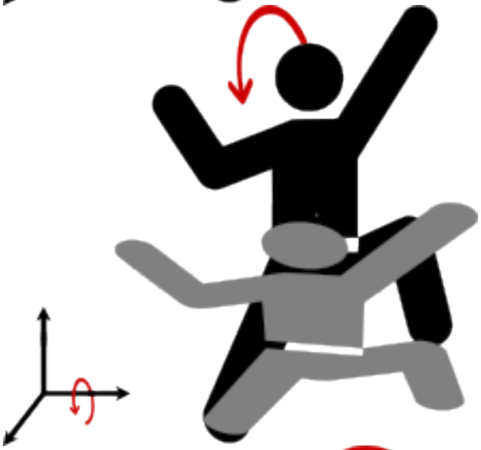

Rotation vers l'arrière

☐ 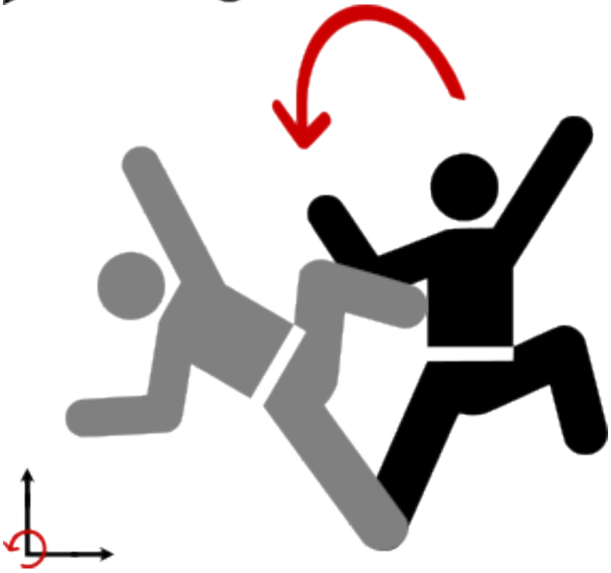

Rotation latérale

☐ 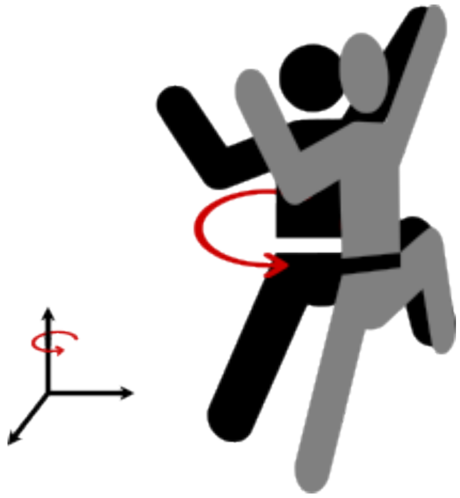 Rotation sur soi

☐ Je ne sais pas

Quelle était la 1ère partie de votre corps à toucher le matelas ?

Groupe principaux	Catégorie	Equivalent OSICS	Légende
Tête et cou	Tête / Face	H	
	Cou / Vertèbre cervicale	N	
Membre supérieur	Epaule / Clavicule	S	
	Bras (partie proximale)	U	
	Coude	E	
	Avant-bras (partie distale)	R	
	Poignet	W	
	Main / Doigts / Pouce	P	
Tronc	Sternum / Côtes / Haut du dos	C,D	
	Abdomen (ventre)	O	
	Bas du dos / Pelvis / Sacrum	B,L	
Membre inférieur	Hanche / Aine	G	
	Cuisse	T	
	Genou	K	
	Jambe / Tendon d'achille	Q,A	
	Cheville	A	
	Pied / Orteils	F	

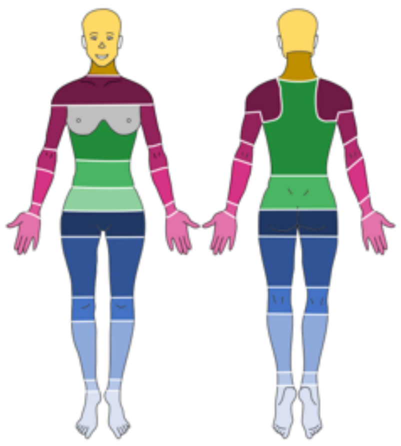

Veuillez sélectionner une seule des propositions suivantes :

◯ (Tête et cou) Tête / Face

◯ (Tête et cou) Cou / Vertèbre cervicale

◯ (Membre supérieur) Epaule / Clavicule

◯ (Membre supérieur) Bras (partie proximale)

◯ (Membre supérieur) Coude

◯ (Membre supérieur) Avant-bras (partie distale)

◯ (Membre supérieur) Poignet

◯ (Membre supérieur) Main / Doigts / Pouce

◯ (Tronc) Sternum / Côtes / Haut du dos

- ◯ (Tronc) Abdomen (ventre)
- ◯ (Tronc) Bas du dos / Pelvis / Sacrum
- ◯ (Membre inférieur) Hanche / Aine
- ◯ (Membre inférieur) Cuisse
- ◯ (Membre inférieur) Genou
- ◯ (Membre inférieur) Jambe / Tendon d'achille
- ◯ (Membre inférieur) Cheville
- ◯ (Membre inférieur) Pied / Orteils
- ◯ (Autre) Je ne sais pas

Parmi les schémas ci-dessous, lequel est le plus représentatif de votre position au moment où vous avez impacté le matelas ?

Veuillez cliquer directement sur l'image pour la sélectionner

Veuillez sélectionner une seule des propositions suivantes :

◯ 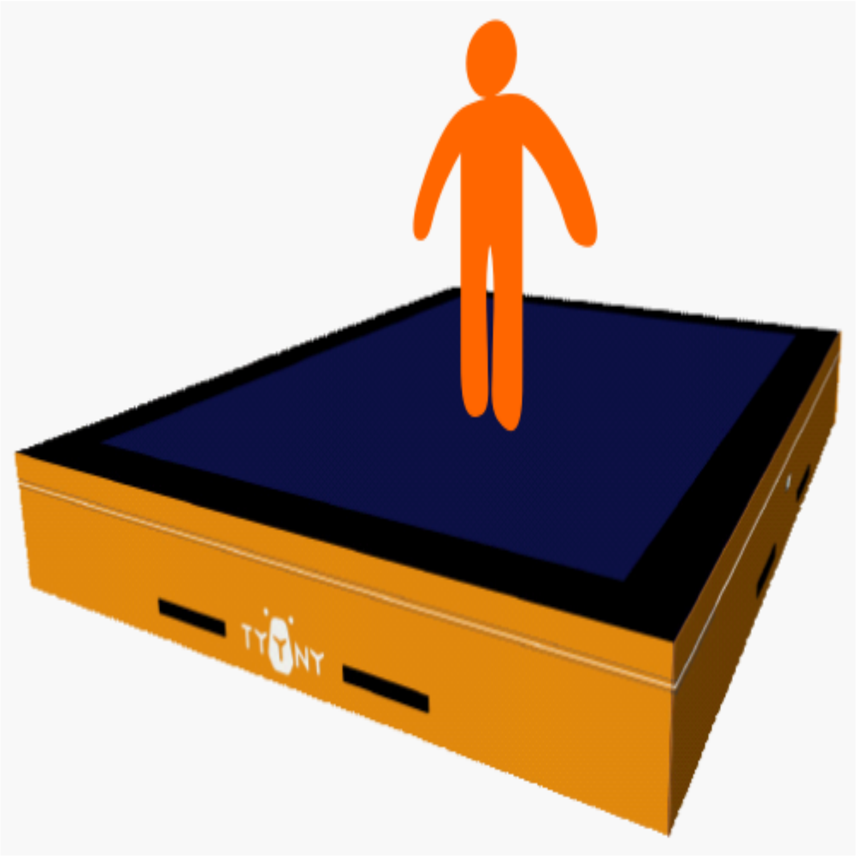

Debout sur les pieds

○ 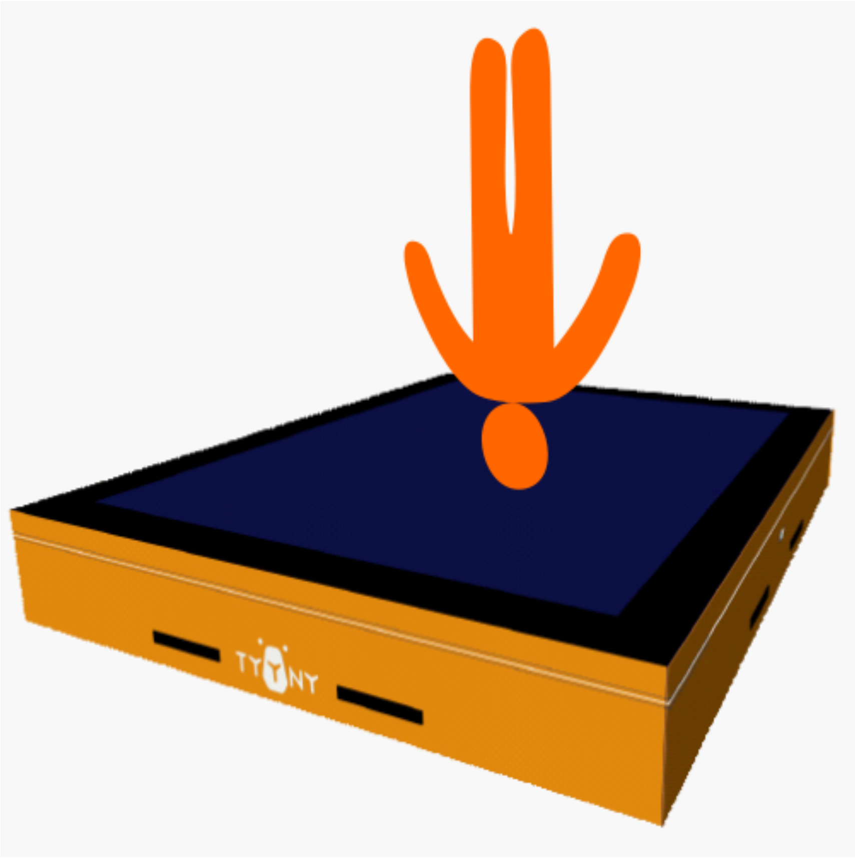 Tout droit la tête en bas

○ 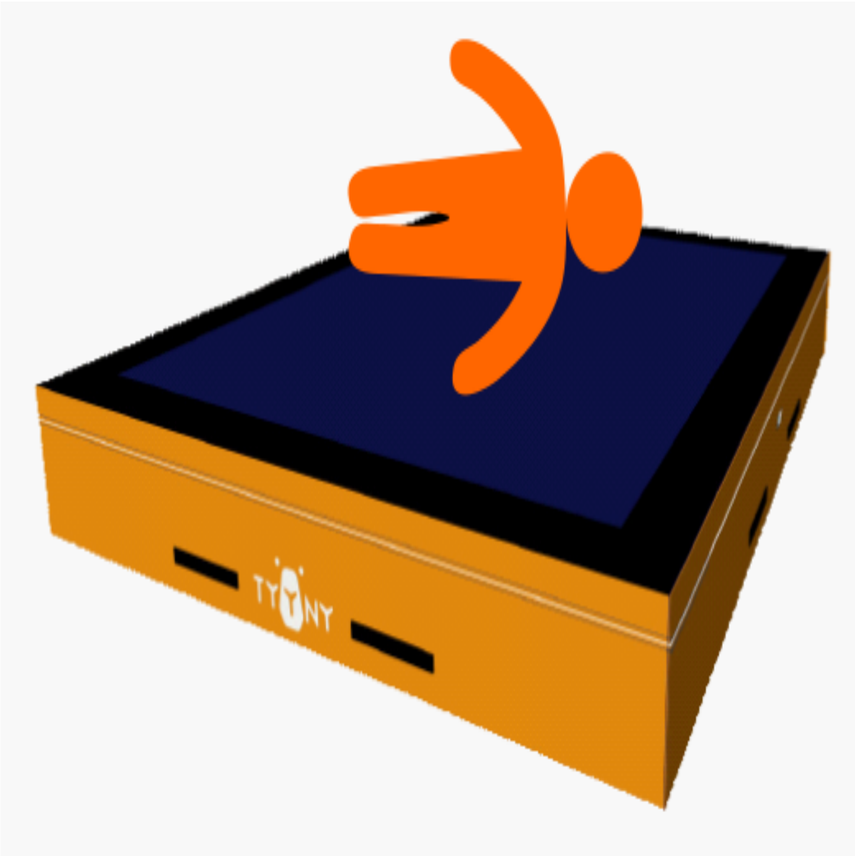 De dos ou de face au tapis

○ 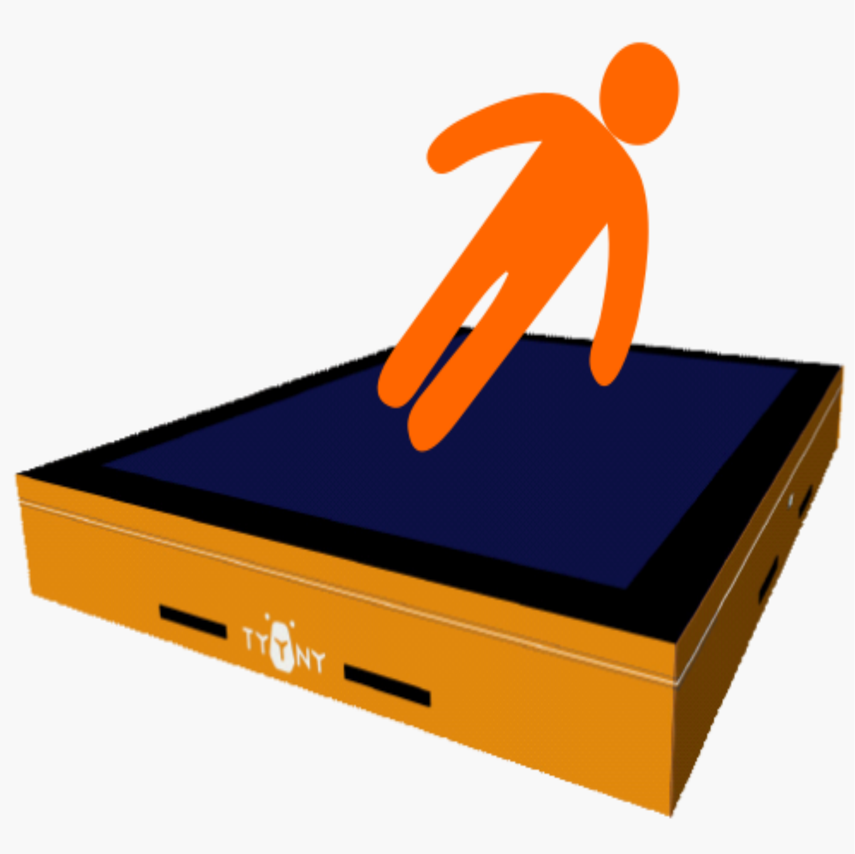 Penché sur le coté les pieds en bas

○ 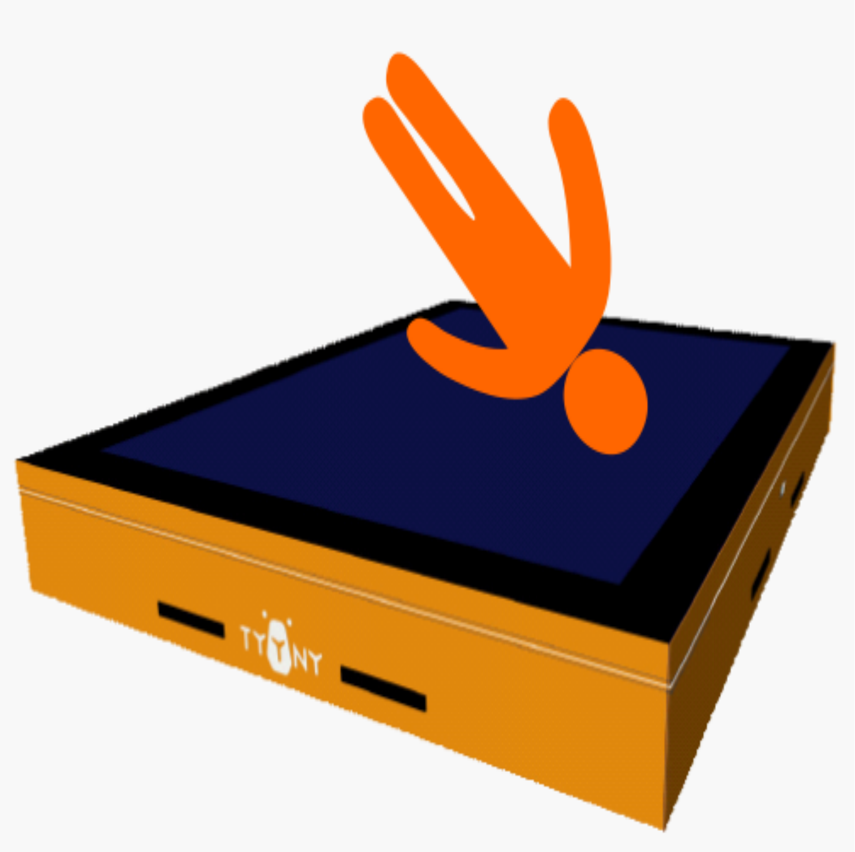 Penché sur le côté la tête en bas

◯ 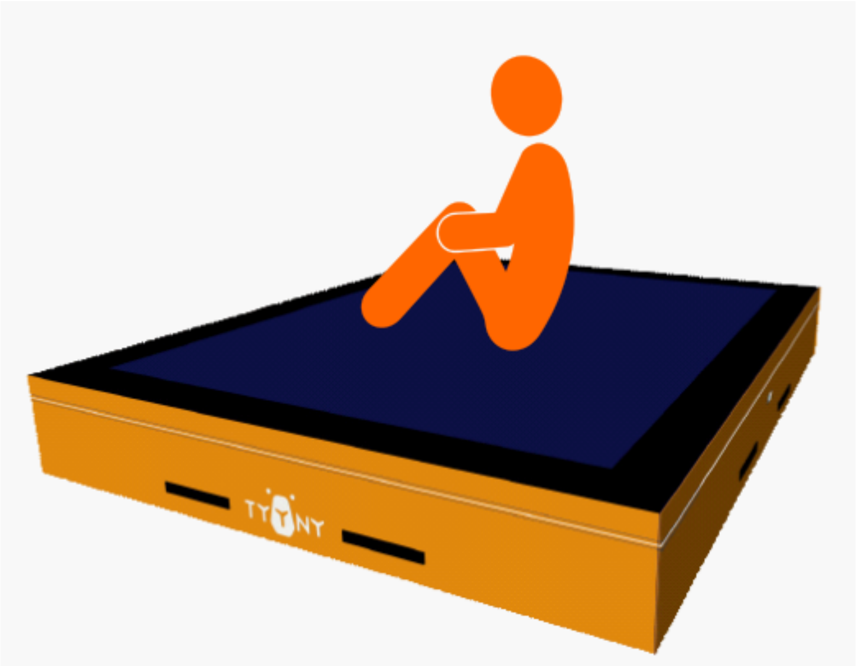 Sur les fesses

◯ 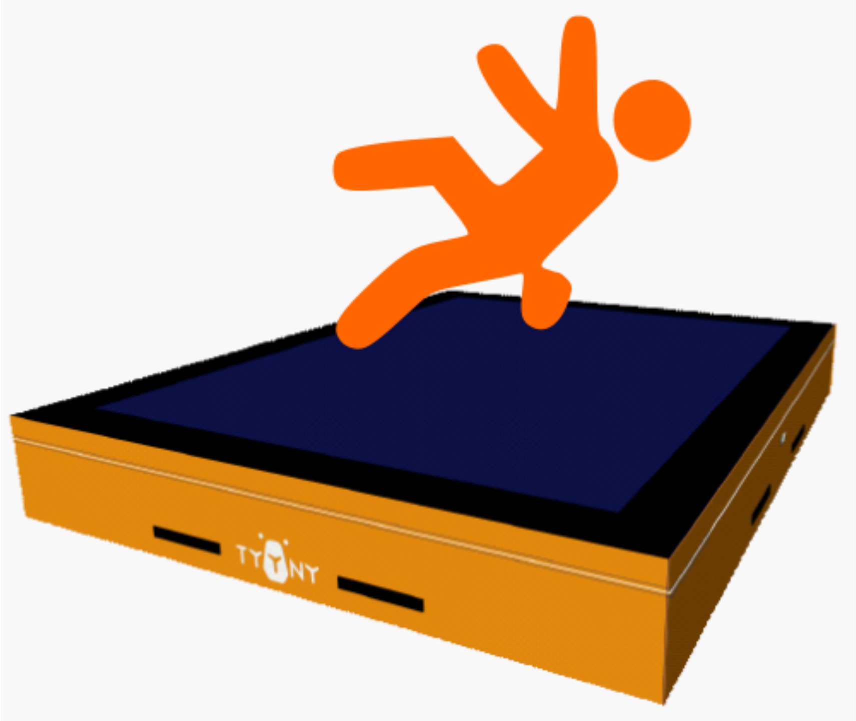 Sur les pieds mais penché en arrière

◯ Je ne suis pas tombé sur un matelas

◯ Je ne sais pas

Vous allez trouver dans les grilles ci-dessous **des actions, situations ou expériences** que vous pouvez parfois vivre dans votre **pratique de l'escalade de bloc**. Indiquez **à quel point vous aimez ou n’aimez pas chacune d’entre-elles**. Sachez qu'aucune réponse n'est juste, elle doit avant tout correspondre le plus possible au **plaisir que vous éprouvez** dans chacune de ces situations.

Dans le cadre de l'escalade de bloc, ce qui me plaît c'est :

Choisissez la réponse appropriée pour chaque élément :

	Pas du tout d'accord	Pas d'accord	Plutôt pas d'accord	Ni en désaccord, ni en accord	Plutôt d'accord	D'accord	Tout à fait d'accord
**Prendre un risque**	○	○	○	○	○	○	○
**Améliorer mes résultats**	○	○	○	○	○	○	○
**Battre mes adversaires**	○	○	○	○	○	○	○
**Aller au-delà de ce que j'ai déjà fait**	○	○	○	○	○	○	○
**Tenter quelque chose au risque de tout perdre**	○	○	○	○	○	○	○
**Être parmi les meilleur·es**	○	○	○	○	○	○	○
**Faire la meilleure performance possible pour moi**	○	○	○	○	○	○	○
**Se mettre en danger**	○	○	○	○	○	○	○
**Tenter un exploit, même s'il y a des risques**	○	○	○	○	○	○	○
**Être meilleur·e que les autres**	○	○	○	○	○	○	○
**De constater que j'ai progressé**	○	○	○	○	○	○	○
**Jouer à me faire peur**	○	○	○	○	○	○	○
**Améliorer mes**							

**propres performances**	○	○	○	○	○	○	○
**Obtenir le meilleur classement possible**	○	○	○	○	○	○	○

Avez-vous déjà subi une ou plusieurs **autre·s blessure·s** à la suite d'une chute en escalade de bloc ?

Veuillez sélectionner une seule des propositions suivantes :

- ○ Oui
- ○ Non

Parmi les zones anatomiques suivantes, indiquer le nombre des **autres blessures** subies pour chacune d'elles.

Groupe principaux	Légende
Tête et cou	
Membre supérieur	
Tronc	
Membre inférieur	

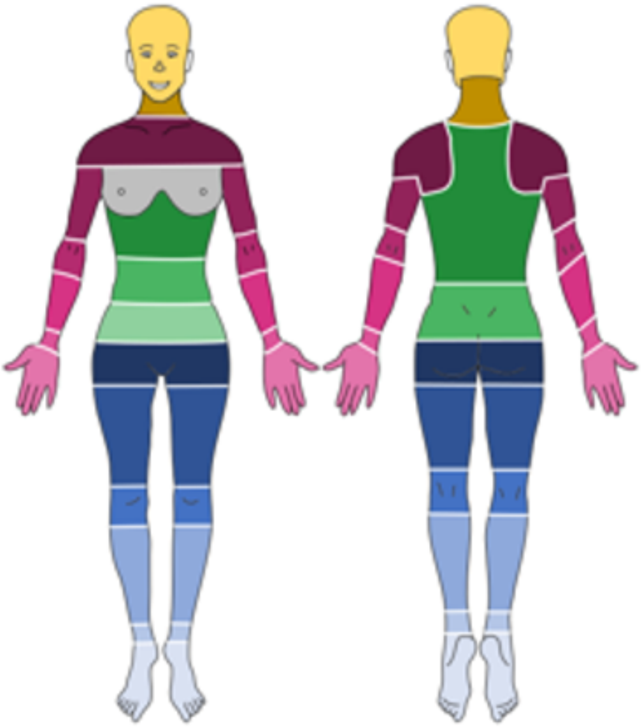

Veuillez écrire votre(vos) réponse(s) ici :

Tête et cou [ ]

Membre supérieur [ ]

Tronc [ ]

Membre inférieur [ ]

Si vous le souhaitez, il est tout à fait possible de remplir ce questionnaire plusieurs fois si vous avez subi plusieurs chutes différentes ayant entrainé des blessures.

Si vous souhaitez nous communiquer des informations supplémentaires concernant votre chute/blessure (une description détaillée de la chute, des détails supplémentaires sur la blessure, le nombre de jours d'arrêt lié à la blessure, etc.), nous vous invitons à les renseigner dans le champ ci-dessous

Veuillez écrire votre réponse ici :

02.09.2024 – 10:52

Envoyer votre questionnaire.
Merci d’avoir complété ce questionnaire.
